# Beyond Deshielding: NMR Evidence of Shielding in Hydridic and Protonic Hydrogen Bonds

**DOI:** 10.1021/acs.jctc.5c00870

**Published:** 2025-07-31

**Authors:** Debashree Manna, Rabindranath Lo, Maximilián Lamanec, Jana Pavlišová, Ondřej Socha, Martin Dračínský, Pavel Hobza

**Affiliations:** † Institute of Organic Chemistry and Biochemistry, 89220 Czech Academy of Sciences, Flemingovo námĕstí 542/2, Prague 160 00, Czech Republic; ‡ IT4Innovations, VŠB-Technical University of Ostrava, 17. listopadu 2172/15, Ostrava-Poruba 708 00, Czech Republic

## Abstract

The red shift of the X–H stretching frequency, with a significant increase in intensity of the corresponding spectral band and a downfield chemical shift of hydrogen (deshielding) in nuclear magnetic resonance (NMR) spectroscopy, has traditionally been used as a criterion for identifying X–H···Y hydrogen bonds (HBs) where X is the hydrogen donor and Y is the acceptor. However, over the past two decades, it has become evident that certain HBs can exhibit a blue shift in the X–H stretching frequency and may also show a decrease in IR intensity, diverging from classical expectations. In this study, we investigate a wide array of HBs, encompassing both red-shifted and blue-shifted systems, as well as protonic and hydridic HB systems. We focus on understanding the underlying electronic conditions behind the reverse chemical shift effects—upfield shifts (shielding) upon HB formation, challenging the view that hydrogen bonding (H-bonding) typically leads to deshielding. We employ state-of-the-art quantum chemical methods, integrating computed NMR shielding tensors and electron deformation density, in combination with experimental NMR, to probe that phenomenon. The computational findings are thoroughly validated against experimental results. Our research confirms that shielding is also possible upon HB formation, thereby broadening the conceptual framework of H-bonding.

## Graphical abstract

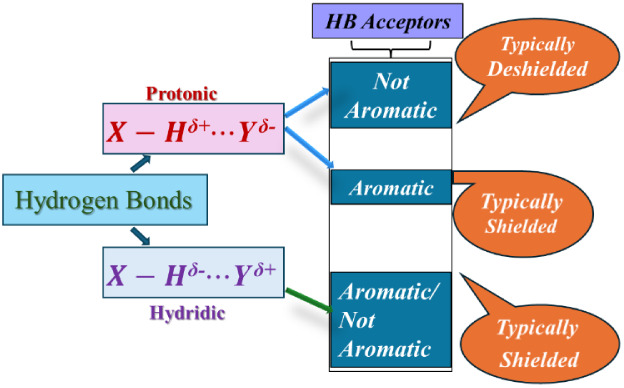

H-bonding remains a highly active and significant area of research across various scientific disciplines. Although the fundamental concept has been established for decades, scientists continue to uncover new aspects and explore innovative applications of hydrogen bonding both theoretically and experimentally.^1–12^ IR and NMR spectroscopy provide direct experimental evidence for the formation of hydrogen bonds (HBs).^13,14^ These techniques have been central to advancing our molecular understanding of H-bonding in gas-phase molecules, liquids, and solid-state structures. As Pimentel and McClellan emphasized in their foundational work, “the IR intensity (of the X–H stretch) and the proton magnetic resonance (down shift of the H resonance) are probably the most sensitive to H-bond formation.”^15^ This sensitivity arises from the subtle yet detectable shifts in bond strength, electron density, and magnetic environment upon HB formation, which leave distinct spectroscopic signatures.

Classical HBs typically exhibit a red shift in the X–H stretching frequency along with a significant increase in IR intensity.^14^ This is because formation of an X–H···Y HB typically weakens and lengthens the covalent X–H bond. This weakening lowers the vibrational frequency of the X–H stretch. A notable increase in IR intensity is due to a significant change in dipole moment after the HB formation. However, in 2000, we published a study entitled “Blue-Shifting Hydrogen Bond,” reporting an unconventional form of H bonding characterized by a blue shift in the X–H stretching frequency.^16^ Additionally, these systems sometimes show a decrease in IR intensity, a behavior that diverges from classical expectations.^17,18^ Notably, the IR band intensity increases for red-shifting and increases or decreases for blue-shifting HBs, offering a distinct spectroscopic fingerprint. Weaker interaction energies often accompany the blue-shifting HBs, but their unique spectroscopic behavior allows them to be distinguished unambiguously. These systems serve as a reminder that H-bonding is not a monolithic phenomenon but exists along a spectrum of structural and electronic variations.

Recently, we extended the frontier of H-bonding by reporting the X–H···Y “hydridic hydrogen bonds”, where hydrogen is covalently bound to an electropositive element, resulting in its partial negative charge, X–H being an electron donor and Y an electron acceptor.^19–21^ This interaction has been termed either a hydride bond, an inverse H-bond, or a charge-inverted H-bond.^22–24^ We adopted the term “hydridic HB” to emphasize both its similarities and differences with classical protonic HBs. This choice underscores the parallels with standard (protonic) HBs—namely, that hydrogen, the lightest element, is covalently bound to a significantly heavier atom. Spectroscopically, these interactions show easily observable red or blue shifts of the X–H stretching frequencies upon HB formation, depending on the nature of the donor and acceptor.

NMR spectroscopy offers another evidence for H-bonding. Typically, the proton magnetic resonance of X–H moves toward a higher chemical shift (downfield) compared to non-hydrogen-bonded X–H.^13,14^ This is the result of strong deshielding of the protons, which is a direct consequence of electron redistribution around the H atom following the HB formation. This downfield shift is commonly used as a spectroscopic ruler for H-bonding.^25^ Moreover, NMR spectroscopy has provided direct evidence for through-H-bond spin–spin coupling between X and Y in a X–H···Y H-bonded system.^26–28^ In our current study, we investigate systematic deviations from these conventional ^1^H NMR patterns for the H-bonded systems. By studying both blue-shifting and red-shifting HBs, including hydridic systems, we identify circumstances under which the expected downfield shift is reversed. These findings suggest that not all HBs follow the same NMR behavior. As our understanding continues to evolve, it seems appropriate to modify the IUPAC definition of HB to captures the full spectrum of H-bonding behavior.

We are aware that, following the IUPAC recommendation, the X-H···Y–Z interaction where H and Y are a hydridic hydrogen and halogen should be named halogen bond (XB), but experimental detection of the XB is difficult. Specifically, the IR detection of the changes of the X–H stretching frequency is unambiguous and easy contrary to the detection of Y–Z stretching frequency. Similarly, the NMR detection of the ^1^H NMR chemical shift provides better evidence on complex formation than the detection of chemical shifts of element Y, which might be poorly sensitive for NMR spectroscopy.

As mentioned earlier, we have considered both protonic and hydridic HBs in this current study. The most common type of HBs are protonic, red-shifted HBs. However, over the last two decades, a considerable number of blue-shifted HBs have also been reported. The first detailed study of blue-shifted HBs involves chloroform···benzene and substituted benzene complexes, where a blue shift in the C–H stretching frequency was confirmed through theory and gas-phase IR spectroscopy.^29^ Thus, to see the NMR pattern, we chose chloroform···benzene (Cl_3_C–H···C_6_H_6_) complex in protonic, blue-shifted category. Here C–H···π type HB is formed. In the protonic, red-shifted category, we chose chloroform···pyridine (Cl_3_C–H···C_5_H_5_N) complex. Importantly, here, C–H···N hydrogen-bonded structure corresponds to the global minimum. Recently, we have seen that hydridic HBs can also exhibit either red or blue shifts in the X–H stretching frequency. We selected highly polarizable (Me_3_Si)_3_SiH as a hydrogen donor and examined its complexes with a wide range of hydrogen-acceptor molecules. From that list, we chose (Me_3_Si)_3_SiH···C_6_F_6_ in the hydridic blue-shifted category, showing a Si–H···π-hole hydridic HB. (Me_3_Si)_3_SiH···C_6_H_5_NO_2_ complex is also included in the hydridic blue-shifted class. It is to be noted that the strongest π-hole is located on the “N’ center of the C_6_H_5_NO_2_.^30^ Consequently, the global minima at the free energy surface of the complex correspond to the structure with the Si–H···π-hole­(N) interaction motif. Notice, however, by investigating the whole surface, we located several local minima that are energetically close to the global minimum. In the hydridic red-shifted category (Me_3_Si)_3_SiH···ICF_3_ complex is studied in detail. All the optimized geometries are presented in Figure 1. The HBs are shown with dotted lines. Table S1 shows the maximum (*V*_s,max_) and minimum (*V*_s,min_) electrostatic potential (ESP) of HB acceptors and HB donors for all the HB complexes mentioned above. ESP maps are demonstrated in Figure S1. Since hydridic HBs were not widely studied, the NMR patterns for a large number of hydridic HB complexes are also explored to establish a general trend (Tables S2 and S3). Aromaticity plays an important role in NMR spectroscopy. To address the impact of aromaticity, we have examined complexes of chloroform and (Me_3_Si)_3_SiH with aromatic compounds (benzene, hexafluorobenzene), non-aromatic compounds [1,3-cyclohexadiene (C_6_H_8_), 1,2,3,4,5,5,6,6-octafluoro-1,3-cyclohexadiene (C_6_F_8_)], and anti-aromatic compounds [cyclooctatetraene­(C_8_H_8_), 1,2,3,4,5,6,7,8-octafluoro-cyclooctatetraene­(C_8_F_8_)].

**Figure 1:**
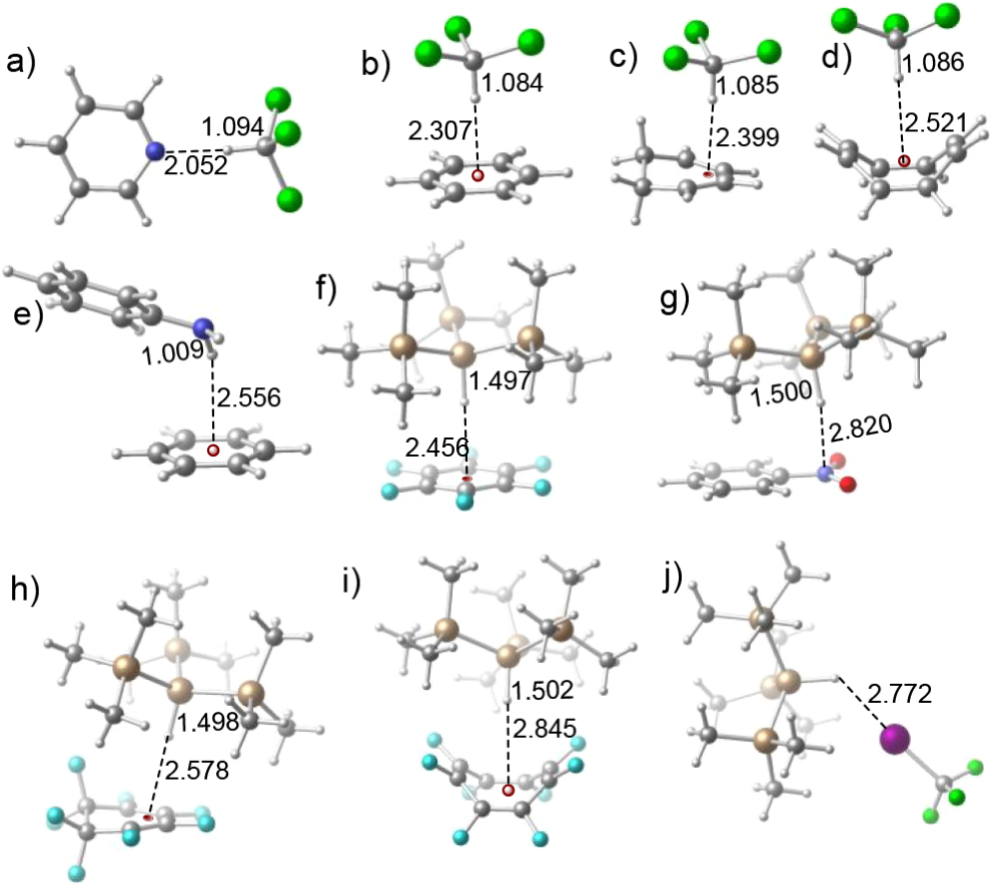
Optimized geometries of (a) Cl_3_C–H···C_5_H_5_N, (b) Cl_3_C–H···C_6_H_6_, (c) Cl_3_C–H···C_6_H_8_, (d) Cl_3_C–H···C_8_H_8_, (e) C_6_H_5_NH_2_···C_6_H_6_, (f) (Me_3_Si)_3_Si–H···C_6_F_6_, (g) (Me_3_Si)_3_Si–H···C_6_H_5_NO_2_, (h) (Me_3_Si)_3_Si–H···C_6_F_8_, (i) (Me_3_Si)_3_Si–H···C_8_F_8_ and (j) (Me_3_Si)_3_Si–H···ICF_3_. The bond distances are in Å. [C: gray, H: white, Cl: green, F: cyan, I: violet, Si: golden, O: red, N: blue, centroids of π systems: dark red].

Table 1 provides data on interaction energies (Δ*E*), Gibbs free energies at 298 K (Δ*G*), NMR shielding values for the monomer and the H-bonded complexes and the difference (Δδ), and IR shift of the X–H stretching frequency for all the complexes in benzene solvent at the PBE0-D3/def2-TZVPP level of theory. We have additionally calculated ΔG from Born–Haber cycle (Scheme S1 in SI) and the values are reported in Table S4. The values from experimental ^1^H NMR are included in Table 1. The COSMO continuum solvent model is used to include the solvent effect.

**Table 1:** Interaction Energy (Δ*E*), Enthalpy (Δ*H*), Gibbs Free Energy (Δ*G*) (in kcal/mol, *T* = 298 K), and Proton Chemical Shifts of HB Donors and Their Complexes, along with Their Respective Differences, Calculated at the PBE0-D3/def2-TZVPP Level of Theory with the COSMO Continuum Solvation Model in Benzene^a^

		Calculated ^1^H chemical shifts^b^		Experimental ^1^H chemical shifts^c^				
	Δ*E*/Δ*H*/Δ*G*	monomer/complex ^1^H δ ppm	Δδ ^1^H ppm	monomer/complex^d^ ^1^H δ ppm	Δδ ^1^H ppm	monomer/complex υ(X–H) cm^–1^	I_COMPLEX_/I_MONOER_	Category
Cl_3_C–H···C_5_H_5_N	–5.33/–4.05/0.62	7.71/11.07	3.36	7.26/8.52	1.26	3179.9/3023.7	250.0	protonic, red, deshielding
Cl_3_C–H···C_6_H_6_	–5.06/–3.78/2.97	7.71/3.90	–3.82	7.26/6.17	–1.09	3179.9/3189.5	25.6	protonic, blue, shielding
Cl_3_C–H···C_6_H_8_	–4.89/–3.56/3.64	7.71/7.98	0.27	7.26/7.17	–0.09	3179.9/3171.6	30.3	nonaromatic HB acceptor
Cl_3_C–H···C_8_H_8_	–5.84/–4.54/2.52	7.71/9.36	1.65	7.26/7.39	0.13	3179.9/3157.14	38.5	antiaromatic HB acceptor
C_6_H_5_NH_2_···C_6_H_6_	–4.20/–2.90/4.35	3.55/1.27	–2.29	3.37/2.79	–0.58	3596.2/3576.6	2.3	protonic, red, shielding
(Me_3_Si)_3_Si–H···C_6_F_6_	–6.76/–5.36/3.43	2.63/–0.08	–2.71	2.10/1.81	–0.29	2126.8/2196.6	0.8	hydridic, blue, shielding
(Me_3_Si)_3_Si–H···C_6_H_5_NO_2_	–5.78/–4.48/4.37	2.63/1.94	–0.69	2.10/2.05	–0.05	2126.8/2155.8	0.9	hydridic, blue, shielding
(Me_3_Si)_3_Si–H···ICF_3_	–4.53/–3.30/5.39	2.63/1.87	–0.77	-	-	2126.8/2077.0	2.8	hydridic, red, shielding
(Me_3_Si)_3_Si–H···C_6_F_8_	–6.23/–4.94/3.60	2.63/2.47	–0.17	-	-	2126.8/2171.9	1.2	nonaromatic HB acceptor
(Me_3_Si)_3_Si–H···C_8_F_8_	–7.63/–6.22/3.83	2.63/2.24	–0.39	-	-	2126.8/2136.4	1.2	antiaromatic HB acceptor

a The IR stretching frequencies (cm^–1^) of X–H for both the complexes and monomers and their intensity ratios between complex and monomer are provided. Experimental ^1^H chemical shifts are also displayed.

b ^1^H NMR of TMS taken as a reference with the isotropic shielding value of 31.506 ppm.

c The ^1^H chemical shifts of chloroform and (Me_3_Si)_3_Si–H in the gas phase, as determined experimentally, are 7.25 and 2.10 ppm, respectively.

d The experimental shifts were obtained for Cl_3_C–H and (Me_3_Si)_3_Si–H dissolved in the interacting partner.

The following equation is used to calculate the interaction energies

1 $$\Delta E=E_{complex}-\left(E_{\left(monomer\;1\right)}+E_{\left(monomer\;2\right)}\right)$$

where, *E* corresponds to the electronic total energies of the fully optimized structures.

The interaction free energies are calculated using the following equation,

2 $$\Delta G=G_{complex}-\left(G_{\left(monomer\;1\right)}+G_{\left(monomer\;2\right)}\right)$$

where *G* denotes the free energies of the fully optimized structures.

Table 1 shows that CHCl_3_···pyridine is the most thermodynamically stable compound with a Δ*G* value of 0.62 kcal/mol among all the HB complexes we considered here. This is also evident from the largest difference between the *V*_s,min_ value of pyridine and the *V*_s,max_ value of the CHCl_3_ (Table S1). The calculated X–H stretching frequency suggests a strong red shift. This complex also shows the shortest hydrogen bond distance of 2.052 Å. As expected, the change in chemical shielding values (Δδ) after hydrogen bond formation indicates significant deshielding in both theoretical calculations and the ^1^H NMR experiment. The blue-shifted CHCl_3_···benzene complex is slightly less stable in terms of Δ*E* (−5.06 kcal/mol), but from the entropy reasons, noticeably less stable in terms of Δ*G* values. In line with the previous reports, this complex shows a blue shift of about 10 cm^–1^.^29^ For this complex, a reversal in chemical shielding values is observed upon HB formation. The negative Δδ was observed in both theory and the experiment, suggesting a strong HB directed shielding effect. This shielding effect is unrelated to the blue-shifting of the HB, as confirmed by analysis of the fluoroform···acetone complex. To date, the largest observed blue shift, 27 cm^–1^, occurs in the fluoroform···acetone-*d*_6_ complex.^17^ Our theoretical calculations yield a positive Δδ value (+1.64 ppm) for this system, indicating that the shielding observed in the CHCl_3_···benzene complex is not a consequence of HB blue-shifting. This observation suggests that the shielding effect may be due to the aromaticity of benzene since the “H” of CHCl_3_ is positioned in the shielding zone of the benzene aromatic ring current. To further understand the effect of aromatic ring current, we considered two other complexes with the C–H···π HB, CHCl_3_···C_6_H_8_ and CHCl_3_···C_8_H_8_. It is important to note that C_6_H_8_ and C_8_H_8_ are classified as nonaromatic and antiaromatic systems with no aromatic ring current. However, as shown in Figure S1, both systems are π-electron rich and can effectively form C–H···π HBs with CHCl_3_. Theoretical as well as experimental NMR data show that the shielding effect is diminished in these complexes, suggesting that the shielding observed in the CHCl_3_···benzene complex arises primarily from the aromatic ring current of benzene. Here, we also report the aniline···benzene complex, in which a hydrogen bond is formed between one amine hydrogen and the π-cloud of benzene. This complex serves as an example of a red-shifting HB that exhibits a shielding effect.

Table 1 also presents data for hydridic HB complexes: the blue-shifted complex (Me_3_Si)_3_Si–H···C_6_F_6_, (Me_3_Si)_3_Si–H···C_6_H_5_NO_2_ and the red-shifted complex (Me_3_Si)_3_SiH···ICF_3_. In addition to these, Tables, S2 and S3 provide computational data for a wide range of hydridic HB complexes. Surprisingly, all the hydridic HBs we investigated exhibit shielding of the hydridic hydrogen upon HB formation (Tables S2 and S3). The theoretical observation is supported by ^1^H NMR experiments performed for the (Me_3_Si)_3_Si–H···C_6_F_6_ and (Me_3_Si)_3_Si–H···C_6_H_5_NO_2_ complexes, which also confirm the shielding of the silane hydrogen after HB formation. Note that chemical shift values obtained from the computational ^1^H NMR for the other local minima structures of (Me_3_Si)_3_Si–H···C_6_H_5_NO_2_ complex are rather deviated from the experimentally observed chemical shift value (Table S5). Furthermore, we have also reported (Me_3_Si)_3_Si–H···C_6_F_8_ and (Me_3_Si)_3_Si–H···C_8_F_8_ complexes where aromatic ring current is broken. Figure S1 suggests that both C_6_F_8_ and C_8_F_8_ systems possess π-holes and can effectively interact with silane “H”. However, for the (Me_3_Si)_3_Si–H···C_6_F_8_ and (Me_3_Si)_3_Si–H···C_8_F_8_ complexes, shielding effects are significantly reduced compared to (Me_3_Si)_3_Si–H···C_6_F_6_ since the aromatic ring current is broken in C_6_F_8_ and C_8_F_8_. It is worth noting that even with six highly electronegative fluorine atoms, perfluorobenzene (C_6_F_6_) maintains an aromaticity equivalent to that of benzene.^31^ The ICSS_*zz*_ isosurfaces of C_6_H_6_ and C_6_F_6_ illustrate significant shielding zones that project out perpendicularly above and below the aromatic ring (Figure S2 and Table S6). The maximum calculated ICSS_*zz*_ values are 30.45 ppm for C_6_H_6_ and 24.20 ppm for C_6_F_6_, suggesting that the out-of-plane π aromaticity of benzene is not significantly greater than that of perfluorobenzene (Table S6). As expected, shielding zones are missing for the nonaromatic (C_6_H_8_, C_6_F_8_) and antiaromatic (C_8_H_8_, C_8_F_8_) systems (Figure S2).

The upfield shift in the ^1^H NMR signal of hydrogen donors upon interaction with the aromatic π systems of C_6_H_6_ and C_6_F_6_ via hydrogen bonding was investigated in more detail using electron deformation density (EDD), and natural bond orbital (NBO) analysis. Upon hydrogen bond formation, a substantial redistribution of electron density is observed for protonic hydrogens, leading to an increase in positive charge (Figure 2). In contrast, hydric hydrogens show a smaller accumulation of electron density upon HB formation (Figures 2 and S3). This suggests that the hydridic HBs upfield chemical shift is due to an increase in electron density on the hydridic hydrogen upon HB formation. Notice, a second H-bonding interaction is also present between the −CH_3_ hydrogen and the oxygen of the -NO_2_ group in the (Me_3_Si)_3_Si–H···C_6_H_5_NO_2_ complex. Furthermore, NBO analysis provides the charge delocalization and conjugative interactions in the H-bonded complexes.

**Figure 2:**
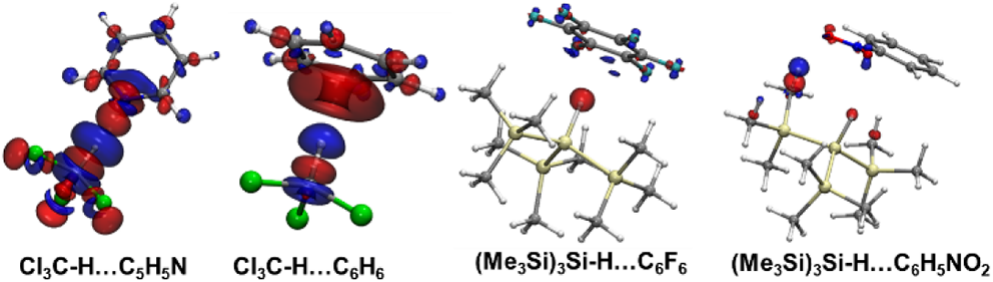
EDD showing charge redistribution upon H-binding formation at isosurface value of 0.0005 au. Red and blue regions indicate the electron density increase and decrease, respectively. [C: gray, H: white, F: cyan, Cl: green,Si: golden].

NBO charges on hydrogen also align with the results from the EDD, as shown in Table S7. The robustness of the NBO analysis was further verified by employing an alternative functional (ωB97XD), basis set (cc-pVTZ), and different solvents (chloroform and dichloromethane). The intermolecular interactions between donor and acceptor orbitals are shown in Figure S4. The stabilization from the second-order perturbation energy (E(2)) is maximum for the chloroform···pyridine complex. For the C–H···π and Si–H···π-hole interactions, E(2), stabilization values are small (Figure S4).

Despite minimal charge redistribution, a significant upfield shift is observed in the ^1^H NMR signal of the hydridic hydrogen in the (Me_3_Si)_3_Si–H···C_6_F_6_ complex. To understand this, we analyzed the shielding tensor to identify the key factors contributing to the shielding. The chemical shift reflects how electrons are distributed around a nucleus and how easily the nucleus can be magnetically perturbed. NMR tensor analysis provides detailed, orientation-dependent information about nuclear magnetic environments in a molecule. Chemical shift (δ) is experimentally measured, while shielding (σ) is theoretically calculated (eq 3).

3 $$\delta =\sigma_{ref}-\sigma_{iso}$$

σ_ref_ is the shielding of a reference compound (like TMS for ^1^H), σ_iso_ is the isotropic shielding of the nucleus of interest. The diagonalization of the shielding tensor σ, leading to its three principal components (σ_11_, σ_22_, σ_33_), can provide information regarding the electronic environment around the nucleus. σ_iso_ is the average of the three principal components of the shielding tensor (eq 4). Each principal shielding component consists of two contributions, diamagnetic and paramagnetic (eq 5). Diamagnetic term, σ_dia_ is always positive (shielding), and the paramagnetic term, σ_para_ is usually negative (deshielding).

4 $$\sigma_{iso}=\frac{1}{3}\left(\sigma_{11}+\sigma_{22}+\sigma_{33}\right)$$

$$\sigma_{ii}=\sigma_{\left(ii,dia\right)}+\sigma_{\left(ii,para\right)},\left(i=1,2,3\right)$$ 5

In the compounds we studied here, the σ_dia_ term is affected more after the HB formation (Table S8). A closer look at the principal shielding components obtained from the natural chemical shielding (NCS) analysis reveals the origin of the shielding and deshielding pattern we observed. The σ_33_ component is aligned along the X–H bond for all the complexes and undergoes significant changes upon HB formation. For the hydridic HB complexes, the other two components (σ_11_, σ_22_) are less affected (Figure 3). The (Me_3_Si)_3_Si–H···C_6_F_6_ complex exhibits maximum changes in the σ_33_ component. Here, smaller changes in deshielding contributions from σ_11_ and σ_22_ and enhanced changes in shielding contributions from the σ_33_ component result in relative shielding of the isotopic ^1^H NMR value. For the Cl_3_C–H···C_6_H_6_ and C_6_H_5_NH_2_···C_6_H_6_ protonic HB complexes, high Δσ_33_ values lead to shielding of the hydrogen. However, for chloroform···pyridine complex, deshielding contributions from Δσ_11_ and Δσ_22_ dominate and result in a significant downfield chemical shift of the hydrogen.

**Figure 3:**
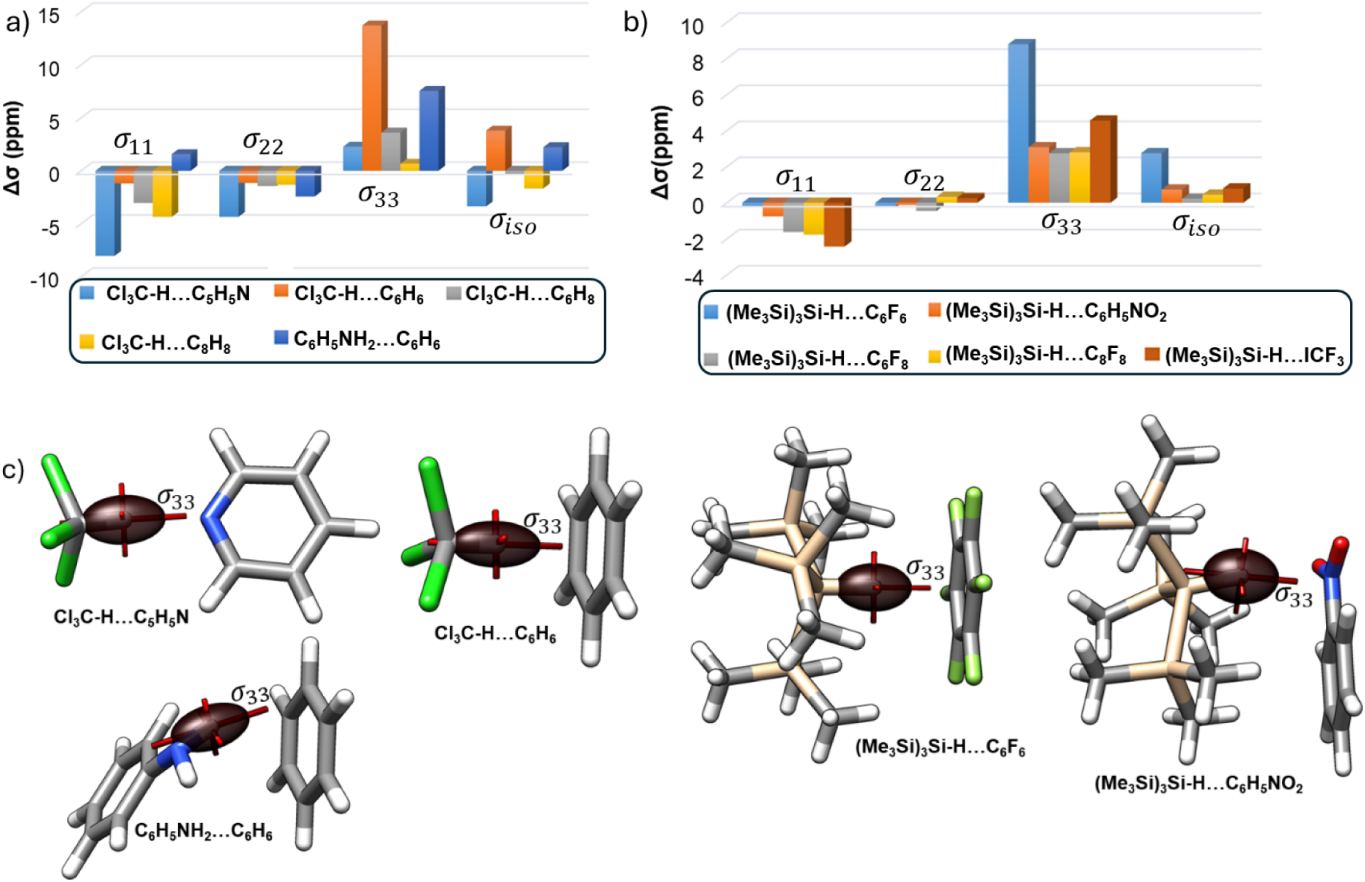
Changes in the σ_11_, σ_22_ and σ_33_ components (Δσ) of H of the X–H upon H-bonding in the (a) protonic and (b) hydridic HBs. (c) Shielding tensors of the H of the X–H bond in selected H-bonded complexes.

These findings underscore the directional nature of H- bonding and highlight the role of electronic structure in modulating the nuclear shielding environment. The observed chemical shifts in the studied complexes result from a combined contribution of changes in the principal components of the chemical shielding tensor. In particular, the σ_33_ component, which is aligned along the X–H bond axis, plays a key role in systems exhibiting a net shielding effect upon hydrogen bond formation. Conversely, in conventional hydrogen-bonded complexes that show a downfield shift (deshielding), the σ_11_ and σ_22_ components dominate the response, reflecting significant perturbations in the perpendicular planes relative to the X–H bond. Additionally, the current density maps for the complexes of CHCl_3_ with aromatic (C_6_H_6_), nonaromatic (C_6_H_8_) and antiaromatic (C_8_H_8_) systems and (Me_3_Si)_3_Si–H with aromatic (C_6_F_6_), nonaromatic (C_6_F_8_) and antiaromatic (C_8_F_8_) systems as presented in Figure S2 provide a visual representation of the distribution of electric current within the complex.

We experimentally measured the ^1^H chemical shifts of chloroform and tris­(trimethylsilyl)­silane, (Me_3_Si)_3_Si–H, in the gas phase and in a range of solvents capable of forming hydrogen-bonding interactions with these molecules. As illustrated in Figure 4, the ^1^H NMR spectrum of neat chloroform exhibits a chemical shift of 7.26 ppm, which is nearly identical to the value observed in the gas phase (7.25 ppm), indicating minimal intermolecular interaction in the neat liquid. In contrast, in benzene solution, the chloroform proton signal is significantly upfield-shifted to 6.17 ppm, consistent with C–H···π interactions in chloroform–benzene intermolecular complexes. Further evidence of such interactions is provided by the temperature dependence of the chloroform ^1^H chemical shift in benzene (Figure 4b). As the temperature increases, the chemical shift gradually moves downfield, toward the gas-phase value. This trend reflects the reduced stability of the intermolecular complexes at higher temperatures, leading to diminished interaction and consequently increased deshielding.

**Figure 4:**
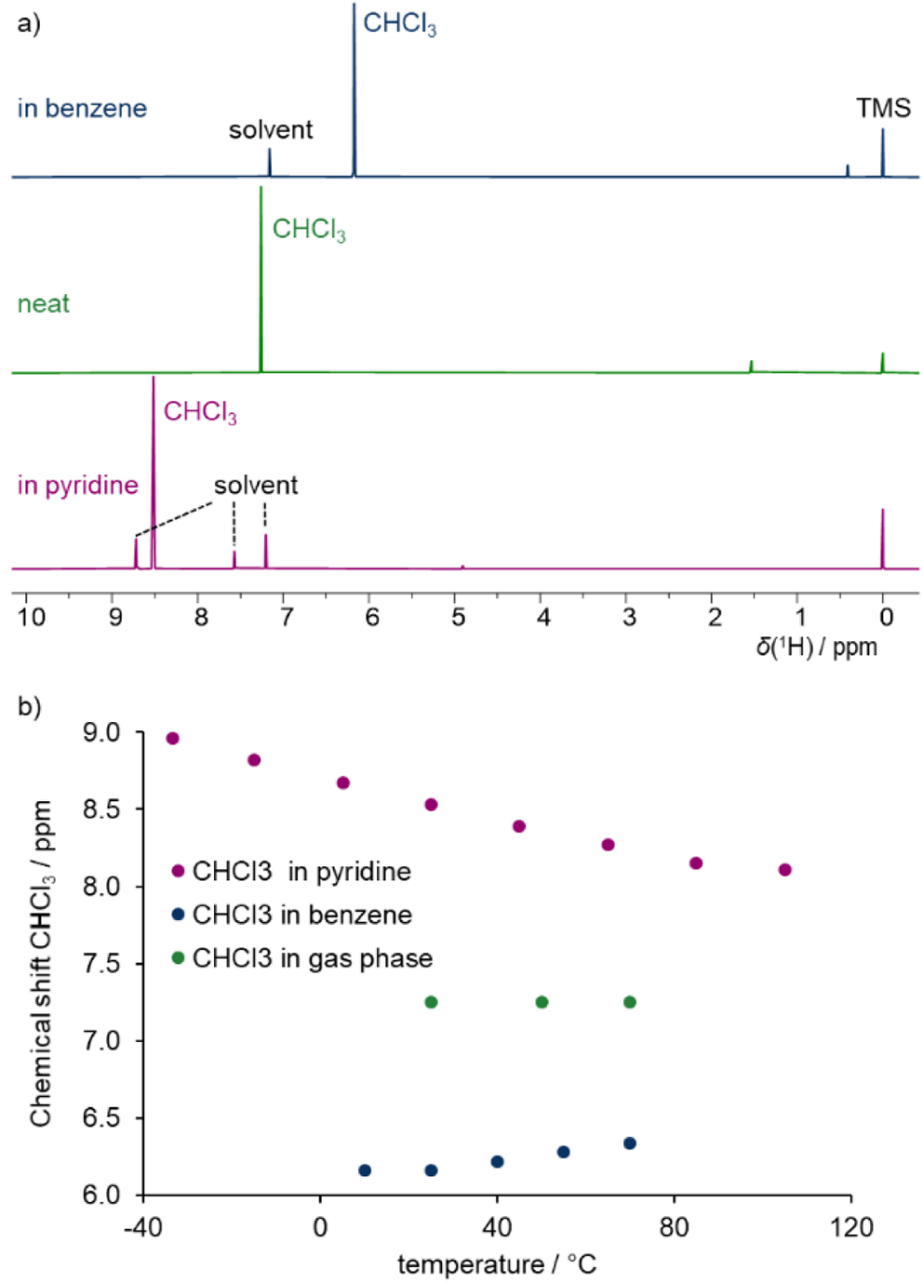
(a) ^1^H NMR spectra of chloroform in C_6_D_6_, CDCl_3_ and pyridine-d_5_. (b) Temperature dependence of chloroform proton chemical shift in these three solvents.

In pyridine, the ^1^H chemical shift of chloroform is observed at 8.52 ppm at room temperature, indicating significant deshielding due to C–H···N hydrogen bonding. Unlike the benzene system, the chemical shift in pyridine decreases with increasing temperature, again suggesting weakening of the complex and a shift toward the gas-phase shielding limit at elevated temperatures. A summary of the measured ^1^H chemical shifts of chloroform in all investigated solvents is provided in Table 1, while additional spectra are available in the Supporting Information (Figures S5, S6 and S7).

For (Me_3_Si)_3_Si–H, the Si–H hydrogen chemical shift in the neat liquid is identical to that in the gas phase (2.10 ppm), indicating negligible intermolecular interactions under these conditions. In contrast, the shift is more upfield (shielded) in both hexafluorobenzene (1.81 ppm) and nitrobenzene (2.05 ppm), suggesting the formation of noncovalent interactions in these solvents (Figure 5). As shown in Figure S8, the Si–H chemical shift in hexafluorobenzene increases with temperature, which supports the interpretation of temperature-dependent decomplexation. At elevated temperatures, the intermolecular interactions are reduced, and the chemical shift approaches the value observed for the isolated molecule. The ^1^H chemical shifts of (Me_3_Si)_3_Si–H are summarized in Table 1, and the spectra are shown in Figure 5.

**Figure 5:**
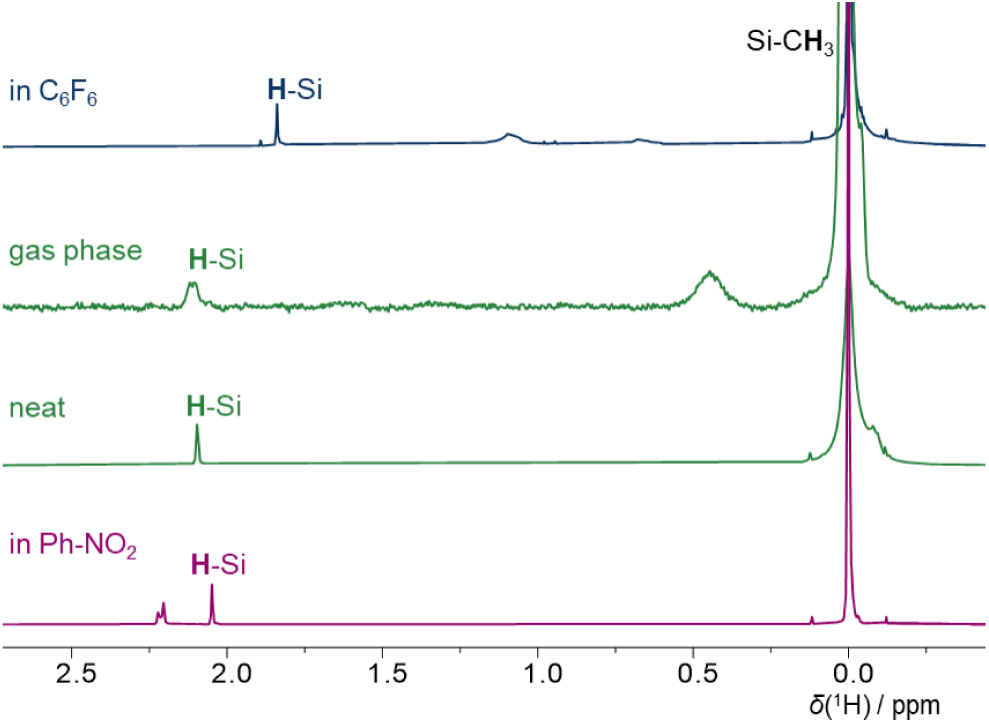
^1^H NMR spectra of (Me_3_Si)_3_SiH in the gas phase and in hexafluorobenzene, in the neat and in nitrobenzene.

In summary, red-shifted and blue-shifted protonic and hydridic HBs were investigated using experimental NMR spectroscopy and computationally.

Protonic HBs usually exhibit a deshielding effect in NMR upon HB formation, but if the HB acceptor is aromatic, and hydrogen is placed in the shielding zone of the aromatic ring, a shielding effect may occur instead. This shielding diminishes when the HB acceptor loses aromaticity or becomes antiaromatic.

Hydridic HBs, on the other hand, typically show shielding, and this effect is amplified by aromaticity in the HB acceptor.

The experimental NMR results show excellent agreement with the computational findings. Additionally, temperature-dependent NMR studies enhance the robustness and resolution of the experimental data presented in this work.

These trends are explained through NMR shielding tensors, EDD, and NBO analysis. The σ_33_ component of the NMR tensor, aligned along the X–H bond, is most strongly perturbed by HB formation. For hydridic HBs, smaller changes in deshielding contributions (σ_11_ and σ_22_) combined with stronger changes in the shielding from the σ_33_ component led to an overall upfield shift in the ^1^H NMR signal. Additionally, electron density depletion is observed on the proton in protonic HBs, whereas hydridic H-bonding shows a modest accumulation of electron density on the hydrogen, reinforcing the shielding trend. Our study reveals that there is no universal relationship between IR frequency shifts and NMR chemical shift behavior of H-Bonded systems. While an apparent inverse correlation between red/blue shifts and shielding can be observed in certain cases, this trend is highly system-dependent and cannot be generalized. The results indicate that the shielding and deshielding effects are more closely tied to the aromaticity of the H acceptor in the HB complexes, as demonstrated in Figure 6.

**Figure 6:**
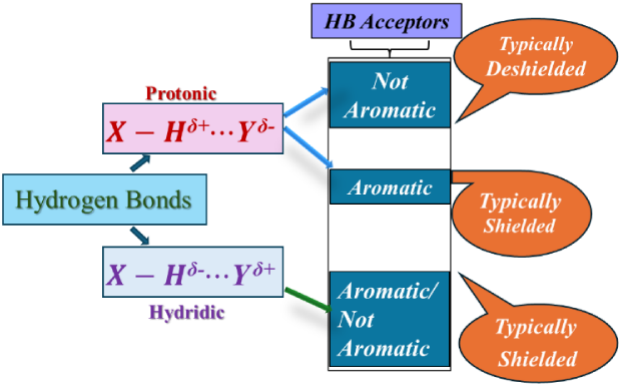
Summary of ^1^H NMR spectra for protonic and hydridic HBs.

We hope that this work contributes to a more complete understanding of the diverse behaviors exhibited by HBs in different chemical environments.

## Supplementary Material



## Notes

Additional tables, figures, experimental details, computational details, and coordinates of optimized geometries (PDF)
