# Supplementary material for: Beyond Deshielding: NMR Evidence of Shielding in Hydridic and Protonic Hydrogen Bonds
Source: J Chem Theory Comput. 2025 Jul 31;21(15):7495–502. doi: 10.1021/acs.jctc.5c00870 (PMC12355688; doi:10.1021/acs.jctc.5c00870)
Supplement: Supplementary file 1 [file ct5c00870_si_001.pdf]

Supporting Information

Beyond Deshielding: NMR Evidence of Shielding in Hydridic and Protonic Hydrogen Bonds

Debashree Manna,[a]‡ Rabindranath Lo,[a]‡ Maximilián Lamanec,[a,b]‡ Jana Pavlišová,[a] Ondřej Socha,[a] Martin Dračínský*[a] and Pavel Hobza*[a,b]

[a] Institute of Organic Chemistry and Biochemistry, Czech Academy of Sciences, Flemingovo náměstí 542/2, 160 00 Prague, Czech Republic;

E-mail: martin.dracinsky@uochb.cas.cz , pavel.hobza@uochb.cas.cz

[b] IT4Innovations, VŠB-Technical University of Ostrava, 17. listopadu 2172/15, 708 00 Ostrava-Poruba, Czech Republic

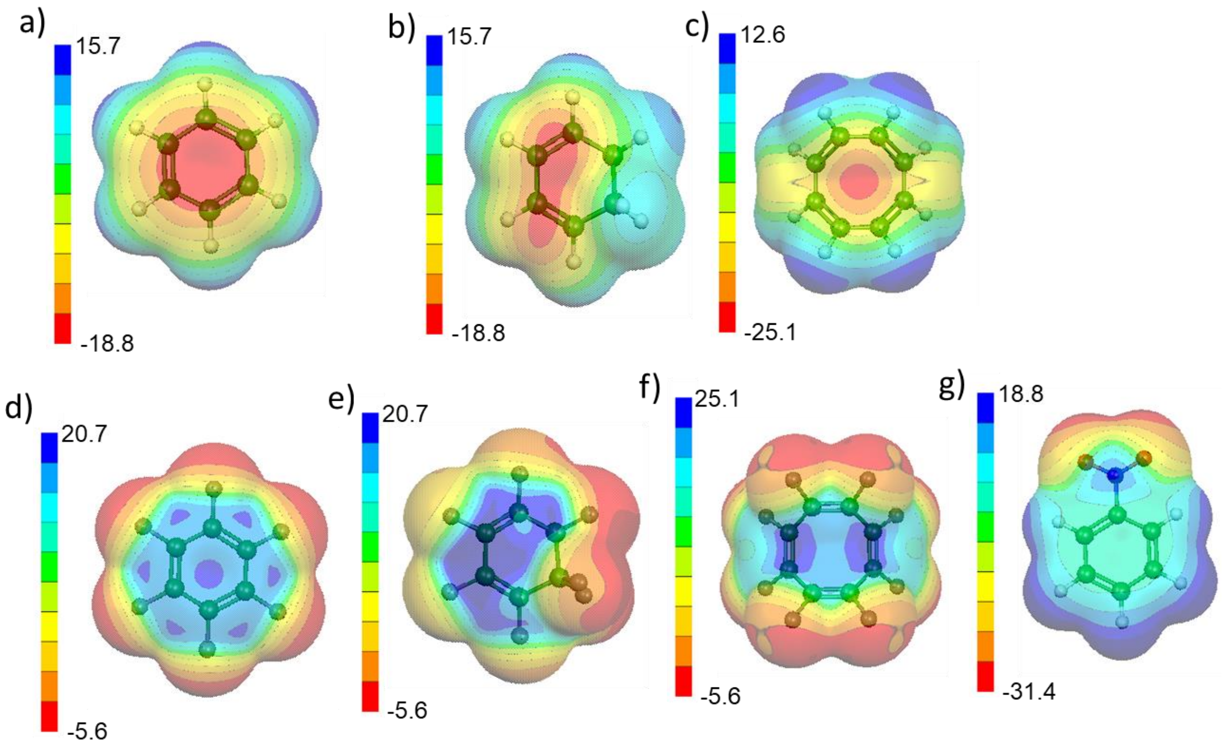

Figure S1. The molecular electrostatic potential (MESP) maps of a) C_6H_6, b) C_6H_8, c) C_8H_8, d) C_6F_6, e) C_6F_8, f) C_8F_8 and g) $C_6H_5NO_2$. The red and blue indicates the negative and positive regions, respectively. The scale is in kcal/mol.

Table S1. The most positive MESP ($V_{s,max}$, kcal/mol) and negative MESP ($V_{s,min}$, kcal/mol) of the studied systems.

	$V_{s,min}$		$V_{s,max}$
C_6H_6	-18.8	C_6F_6	20.7
C_6H_8	-18.0	C_6F_8	31.1
C_8H_8	-24.1	C_8F_8	25.9
		$C_6H_5NO_2$	14.4
C_5H_5N	-40.2	$CHCl_3$	37.8
$(Me_3Si)_3Si$-H	-9.2	ICF_3	33.3

Table S2. The interaction energy (ΔE), Gibbs free energy (ΔG) (in kcal/mol, T = 298 K), and chemical shifts[a] of HB donor ($(Me_3Si)_3SiH$) and hydridic HB complexes, along with their respective differences, calculated at the PBE0-D3/def2-TZVPP level of theory with the COSMO continuum solvation model in toluene. The IR stretching frequencies (cm^{-1}) of X-H for both the complexes and monomers and their intensity ratios are provided.

Systems	ΔE/ΔG	monomer/complex ^{1}H δ ppm	Δδ ^{1}H ppm	monomer/complex υ(X-H) cm^{-1}	Δυ(X-H) cm^{-1}	$I_{COMPLEX}$/ I_{MONOER}
$(Me_3Si)_3SiH\cdots BF_3$	-4.18/7.08	2.66/2.07	-0.59	2127.7/2114.9	-12.8	2.4
$(Me_3Si)_3SiH\cdots BrCN$	-4.28/3.43	2.66/1.96	-0.70	2127.7/2093.0	-34.7	2.4
$(Me_3Si)_3SiH\cdots BrCF_3SO_2$	-5.00/8.54	2.66/2.11	-0.55	2127.7/2082.4	-45.3	3.0
$(Me_3Si)_3SiH\cdots ICN$	-6.21/2.17	2.66/1.53	-1.13	2127.7/2052.3	-75.4	4.5
$(Me_3Si)_3SiH\cdots P(CN)_3$	-8.11/5.60	2.66/2.10	-0.56	2127.7/2046.1	-81.7	8.8
$(Me_3Si)_3SiH\cdots S(CN)_2$	-5.44/7.43	2.66/1.99	-0.67	2127.7/2100.7	-27.0	2.8
$(Me_3Si)_3SiH\cdots C_6ClF_5$	-7.25/6.59	2.66/-0.08	-2.74	2127.7/2202.3	74.6	0.7
$(Me_3Si)_3SiH\cdots C_6BrF_5$	-6.58/7.13	2.66/0.04	-2.62	2127.7/2202.39	74.7	0.7
$(Me_3Si)_3SiH\cdots C_6(CN)_3H_3$	-7.77/6.35	2.66/-0.80	-3.46	2127.7/2208.8	81.1	0.9
$(Me_3Si)_3SiH\cdots C_6(CN)_4H_2$	-8.47/5.75	2.66/-0.76	-3.42	2127.7/2196.2	68.5	1.1
$(Me_3Si)_3SiH\cdots C_6(CN)_6$	-10.60/4.10	2.66/-0.91	-3.57	2127.7/2185.5	57.8	1.6
$(Me_3Si)_3SiH\cdots (CF_3)_3C_3N_3$	-8.16/7.33	2.66/-0.80	-3.46	2127.7/2173.2	45.5	1.6
$(Me_3Si)_3SiH\cdots COF_2$	-3.35/8.16	2.66/2.29	-0.37	2127.7/2131.9	4.2	1.5
$(Me_3Si)_3SiH\cdots NO_2F$	-2.95/8.60	2.66/2.45	-0.21	2127.7/2135.1	7.4	1.2
$(Me_3Si)_3SiH\cdots C_2\ (CN)_4$	-6.64/6.72	2.66/2.14	-0.52	2127.7/2146.5	18.8	3.8
$(Me_3Si)_3SiH\cdots XeF_4$	-4.93/6.92	2.66/2.38	-0.28	2127.7/2133.2	5.5	1.8

[a] ^{1}H NMR of TMS taken as a reference with the isotropic shielding value of 31.528 ppm.

Table S3. The interaction energy (ΔE), free energy (ΔG) (in kcal/mol, T = 298 K), and chemical shifts[a] of HB donor (Me_3SiH) and hydridic HB complexes, along with their respective differences, calculated at the PBE0-D3/def2-TZVPP level of theory with the COSMO continuum solvation model in toluene. The IR stretching frequencies (cm^{-1}) of X-H for both the complexes and monomers and their intensity ratios are provided.

Systems	ΔE/ΔG	monomer/complex ^{1}H δ ppm	Δδ ^{1}H ppm	monomer/complex υ(X-H) cm^{-1}	Δυ(X-H) cm^{-1}	$I_{COMPLEX}$/ I_{MONOER}
$Me_3SiH\cdots BF_3$	-3.36/7.01	4.51/4.32	-0.19	2162.8/2125.4	-37.4	1.9
$Me_3SiH\cdots BrCN$	-2.81/3.63	4.51/4.03	-0.48	2162.8/2116.9	-45.8	1.9
$Me_3SiH\cdots C_6F_6$	-4.29/7.77	4.510/2.60	-1.91	2162.8/2172.4	9.7	1.0
$Me_3SiH\cdots C_6(CN)_3H_3$	-4.47/7.41	4.51/2.03	-2.48	2162.8/2171.3	8.5	1.1
$Me_3SiH\cdots COF_2$	-2.56/8.16	4.51/4.30	-0.21	2162.8/2145.0	-17.7	2.4
$Me_3SiH\cdots ICF_3$	-3.13/7.52	4.51/3.89	-0.62	2162.8/2105.3	-57.4	2.4
$Me_3SiH\cdots ICN$	-4.33/2.72	4.51/3.72	-0.79	2162.8/2071.4	-91.4	3.6
$Me_3SiH\cdots NO_2F$	-2.18/8.74	4.51/4.35	-0.16	2162.8/2153.3	-9.4	1.2
$Me_3SiH\cdots P(CN)_3$	-5.94/6.76	4.51/4.44	-0.07	2162.8/2052.9	-109.8	4.7
$Me_3SiH\cdots S(CN)_2$	-4.02/7.77	4.51/4.02	-0.49	2162.8/2112.5	-50.3	2.3
$Me_3SiH\cdots XeF_4$	-3.48/7.54	4.51/4.42	-0.09	2162.8/2141.9	-20.9	1.5
$Me_3GeH\cdots BF_3$	-3.18/7.24	4.38/4.09	-0.29	2080.1/2036.9	-43.2	1.7
$Me_3GeH\cdots BrCN$	-2.70/3.78	4.38/3.83	-0.55	2080.1/2029.8	-50.4	1.8

$Me_3GeH\cdots C_6F_6$	-4.35/7.86	4.38/2.53	-1.85	2080.1/2085.6	5.5	1.0
$Me_3GeH\cdots C_6(CN)_3H_3$	-4.69/7.56	4.38/1.92	-2.46	2080.1/2085.0	4.8	1.0
$Me_3GeH\cdots COF_2$	-2.50/8.35	4.38/4.12	-0.26	2080.1/2058.4	-21.7	1.3
$Me_3GeH\cdots ICF_3$	-3.07/7.63	4.38/3.70	-0.68	2080.1/2018.0	-62.2	2.3
$Me_3GeH\cdots ICN$	-4.21/2.91	4.38/3.47	-0.91	2080.1/1980.3	-99.8	3.4
$Me_3GeH\cdots NO_2F$	-2.16/8.78	4.38/4.19	-0.19	2080.1/2068.1	-12.1	1.2
$Me_3GeH\cdots P(CN)_3$	-5.94/6.94	4.38/4.21	-0.17	2080.1/1955.3	-124.9	4.5
$Me_3GeH\cdots S(CN)_2$	-3.95/7.90	4.38/3.80	-0.58	2080.1/2023.9	-56.2	2.2
$Me_3GeH\cdots XeF_4$	-3.38/7.62	4.38/4.22	-0.16	2080.1/2053.8	-26.3	1.5
$Me_3SnH\cdots BF_3$	-3.51/6.87	4.75/4.48	-0.27	1878.3/1828.8	-49.5	1.7
$Me_3SnH\cdots BrCN$	-2.88/3.55	4.75/4.21	-0.54	1878.3/1824.1	-54.2	1.8
$Me_3SnH\cdots C_6F_6$	-4.58/7.95	4.75/3.05	-1.70	1878.3/1881.8	3.5	1.0
$Me_3SnH\cdots C_6(CN)_3H_3$	-4.99/7.73	4.75/2.51	-2.24	1878.3/1879.9	1.6	1.0
$Me_3SnH\cdots COF_2$	-2.65/8.02	4.75/4.48	-0.27	1878.3/1853.1	-25.2	1.1
$Me_3SnH\cdots ICF_3$	-3.39/7.58	4.75/4.11	-0.64	1878.3/1807.9	-70.4	2.1
$Me_3SnH\cdots ICN$	-4.79/2.36	4.75/3.84	-0.91	1878.3/1767.9	-110.5	3.3
$Me_3SnH\cdots NO_2F$	-2.31/8.84	4.75/4.57	-0.18	1878.3/1864.0	-14.3	1.1
$Me_3SnH\cdots P(CN)_3$	-6.81/6.36	4.75/4.78	0.03	1878.3/1726.0	-152.3	4.3
$Me_3SnH\cdots S(CN)_2$	-4.32/7.52	4.75/4.15	-0.60	1878.3/1816.7	-61.7	2.3
$Me_3SnH\cdots XeF_4$	-3.69/7.07	4.75/4.66	-0.09	1878.3/1851.1	-27.2	1.3

[a] ^{1}H NMR of TMS taken as a reference with the isotropic shielding value of 31.528 ppm.

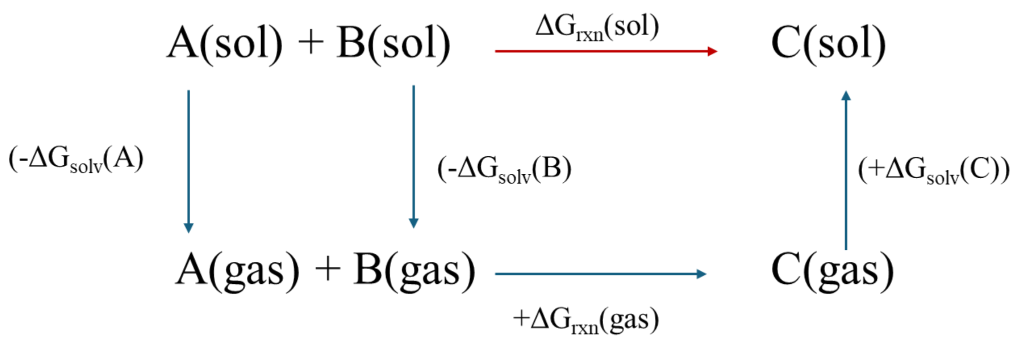

Scheme S1 Thermodynamic cycle for the calculations of ΔG.

$$\Delta G_{rxn}(sol) = \Delta G_{rxn}\ (gas) - \Delta G_{solv}\ (A) - \Delta G_{solv}\ (B) + \Delta G_{solv}\ (C) \qquad (1)$$

$$\Delta G_{rxn}(sol) = \Delta G_{rxn}\ (gas) - \Delta G^{sp}{}_{solv}\ (A) - \Delta G^{sp}{}_{solv}\ (B) + \Delta G^{sp}{}_{solv}\ (C) + \Delta G_{corr} \qquad (2)$$

In addition to ΔG calculated from an optimization-frequency calculation in a continuum solvation model we have additionally calculated ΔG from Born-Haber cycle (scheme S1) using equation (1) and (2). It is worth noting that when all the terms in eqn (1) are evaluated at the *same* level of theory, the resulting solution phase reaction energy is the same as that directly computed within a continuum solvation model at that level of theory. In other words, the solution phase free energy is exactly identical to what would be obtained from an optimization-frequency calculation in a continuum solvation model.[1] However, in equ (2) ΔG_{rxn}(gas) is the Gibbs free energy

change of the reaction in the gas phase and combined with the difference in solvation free energies ($\Delta G^{sp}solv$) between products and reactants. ΔGcorr is the correction associated to the change in standard state from gas phase (1 atm) to solution (1 mol/L) and its value at 298.15 K is 1.89 kcal/mol.[2] In equ (1) ΔGsolv values are obtained via optimisation-frequency calculation in a continuum in solvent whereas for equ (2) gas-phase optimised geometries are used followed by single point calculation in solvent. The ΔG values calculated using the above equations are reported in Table S4.

Table S4. The calculated ΔG values from the thermodynamic cycle.

	ΔG (kcal/mol)	
	Equation 1	Equation 2
$Cl_3C–H\cdots C_5H_5N$	0.62	3.37
$Cl_3C–H\cdots C_6H_6$	2.97	5.09
$Cl_3C–H\cdots C_6H_8$	3.64	5.66
$Cl_3C–H\cdots C_8H_8$	2.52	4.34
$C_6H_5NH_2\cdots C_6H_6$	4.35	6.60
$(Me_3Si)_3Si–H\cdots C_6F_6$	3.43	4.48
$(Me_3Si)_3Si–H\cdots C_6H_5NO_2$	4.37	6.91
$(Me_3Si)_3Si–H\cdots ICF_3$	5.39	6.38
$(Me_3Si)_3Si–H\cdots C_6F_8$	3.60	7.32
$(Me_3Si)_3Si–H\cdots C_8F_8$	3.83	5.86

Table S5. The proton chemical shifts of $(Me_3Si)_3Si–H$ and various local minima structures of $(Me_3Si)_3Si–H\cdots C_6H_5NO_2$ complex, along with their respective differences, calculated at the PBE0-D3/def2-TZVPP level of theory with the COSMO continuum solvation model in benzene.

	Calculated ^1H chemical shifts[a]	
	monomer/complex ^1H δ ppm	Δδ ^1H ppm
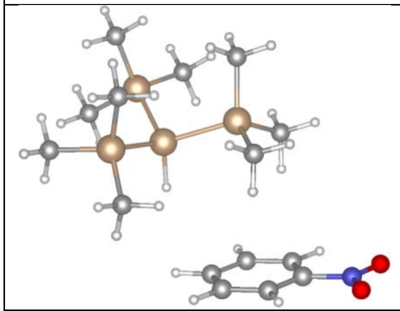	2.63/2.91	0.28

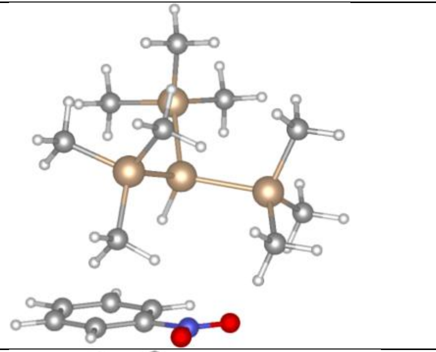	2.63/1.50	-1.13
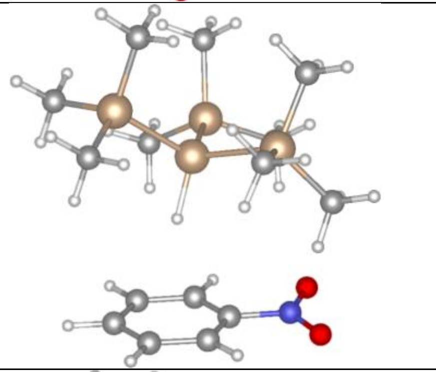	2.63/0.04	-2.59
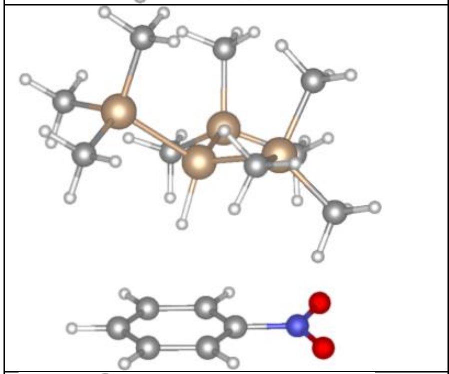	2.63/0.04	-2.59
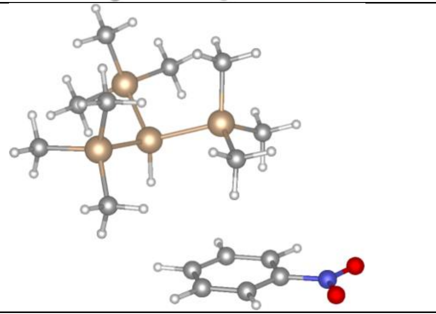	2.63/2.91	0.28

[a] ^{1}H NMR of TMS taken as a reference with the isotropic shielding value of 31.506 ppm.

Table S6. The maximum and minimum Z-component of magnetic shielding ($ICSS_{zz}$) in ppm.

	Maximum	Minimum
C_6H_6	30.45 (0.96, -0.96 Å)	16.45 (0.0 Å)
C_6F_6	24.20 (0.82, -0.82 Å)	18.28 (0.0 Å)

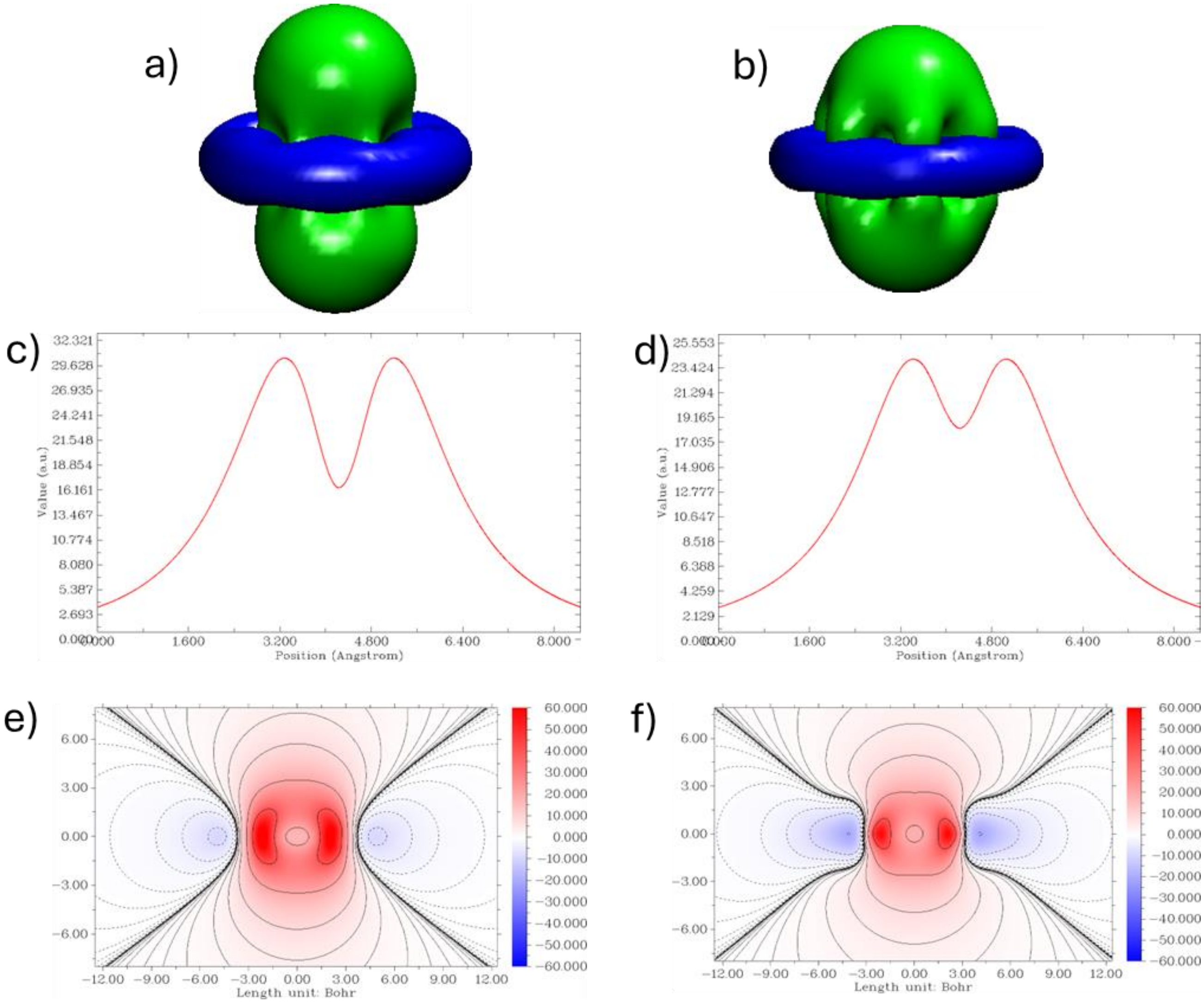
a)
b)
c)
d)
e)
f)
Value (a.u.)
Position (Angstrom)
Length unit: Bohr

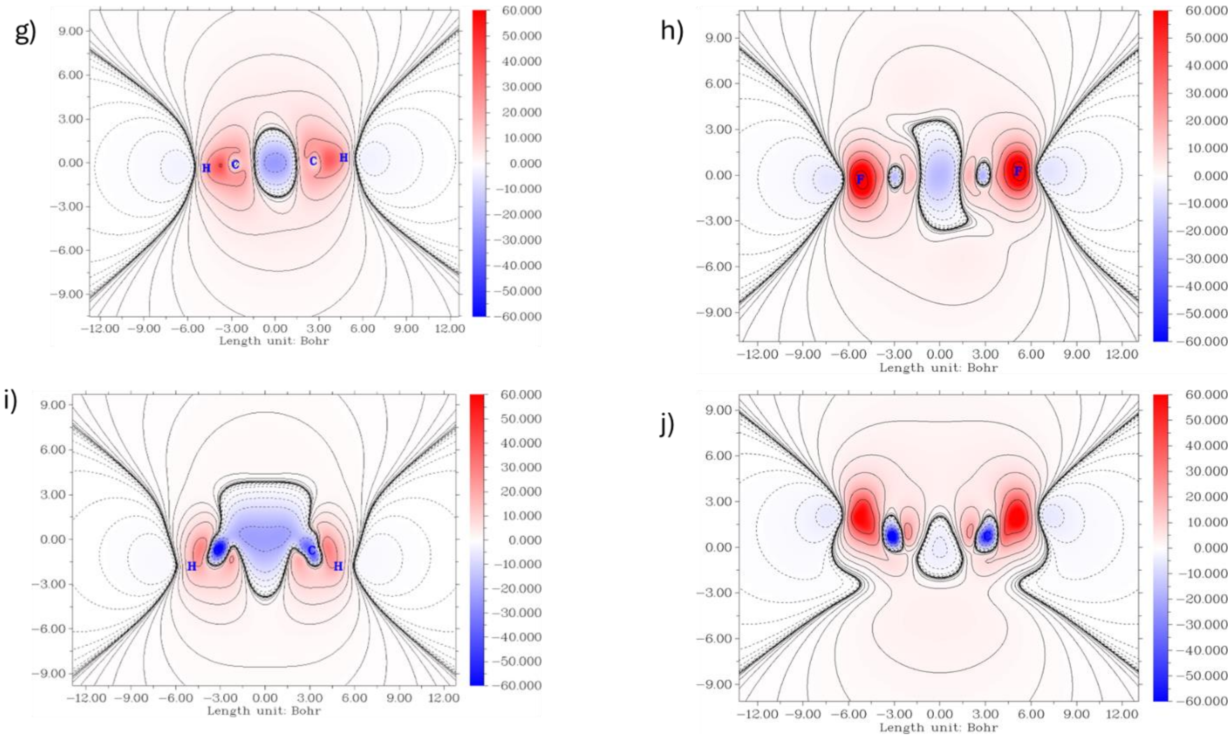

Figure S2. The isosurface of $ICSS_{zz}$ of a) benzene, b) perfluorobenzene, maximum of Z-component of magnetic shielding of c) benzene, d) perfluorobenzene, color-filled contour map of $ICSS_{ZZ}$ in the slice plane perpendicular to the ring of e) C_6H_6, f) C_6F_6, g) C_6H_8, h) C_6F_8, i) C_8H_8, j) C_8F_8. The color scale is given in ppm.

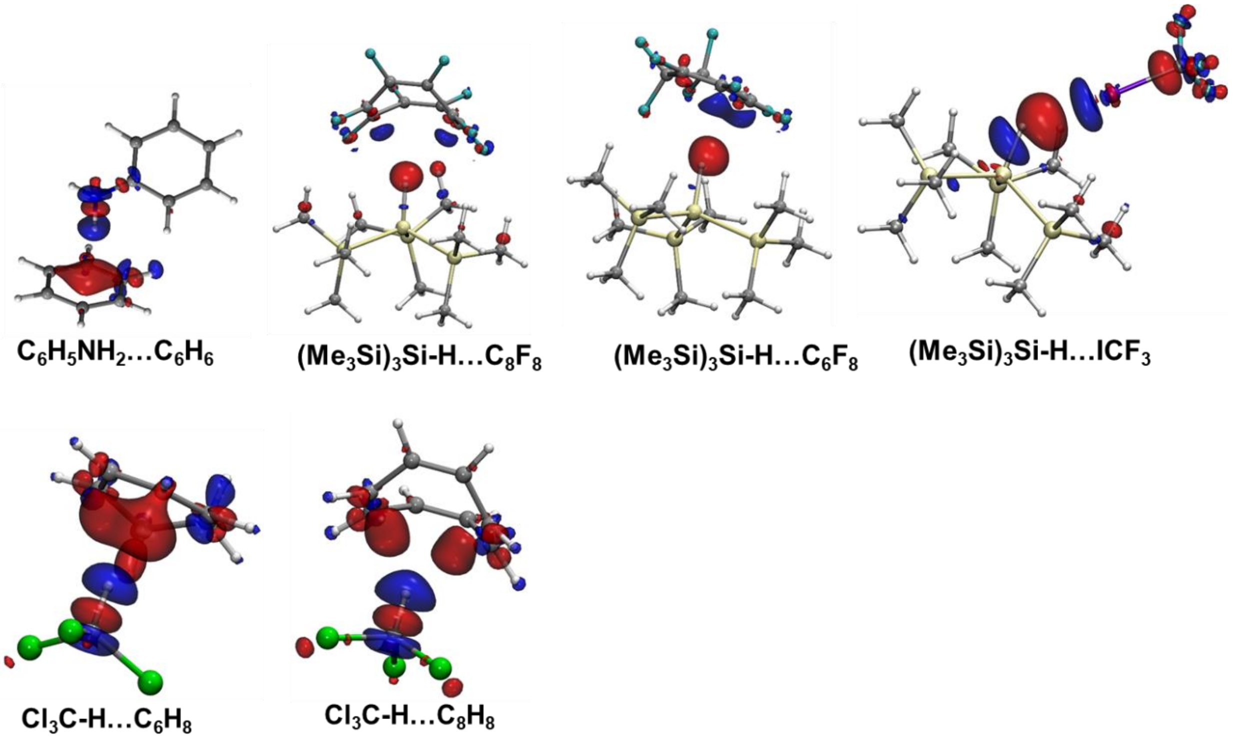

Figure S3. Electron deformation density (EDD) showing charge redistribution upon H-binding formation. Red and blue regions indicate the electron density increase and decrease respectively. [C:grey, H:white, F:cyan, Cl: green,Si:golden, I:violet]

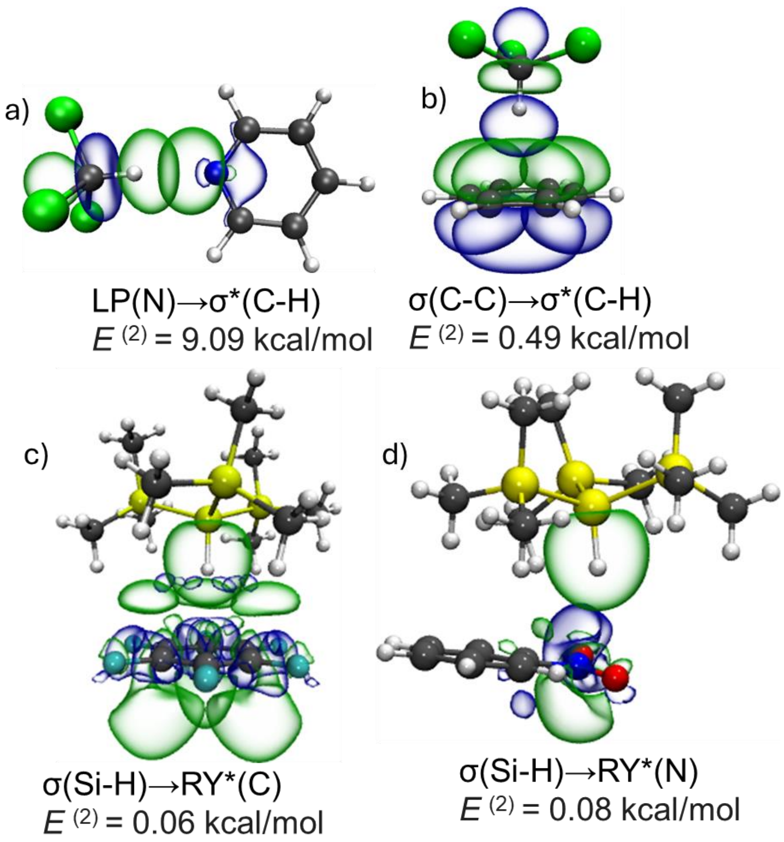

Figure S4. The interacting donor and acceptor orbitals of a) Cl_3C-H...C_5H_5N, b) Cl_3C-H...C_6H_6, c) $(Me_3Si)_3Si$-H…C_6F_6 and d) $(Me_3Si)_3Si$-H…$C_6H_5NO_2$. The second-order perturbation energy $E^{(2)}$ values are given. [C:grey, H:white, Cl: green, Si:yellow, N:blue, O:red]

Table S7. The NBO charges on hydrogen atom in the H-bonded complexes. The charges for the isolated systems given in parenthesis.

Complexes	Medium	NBO charges on H atom		
		PBE0-D3/def2-TZVPP	PBE0-D3/cc-pVTZ	wB97XD/def2-TZVPP
Cl_3C-H... C_5H_5N	benzene	0.292 (0.247)	0.292 (0.232)	0.285 (0.243)
	chloroform	0.294 (0.254)	0.295 (0.239)	0.288 (0.250)
	dichloromethane	0.296 (0.258)	0.296 (0.242)	0.289 (0.254)
Cl_3C-H...C_6H_6	benzene	0.262	0.242	0.255
	chloroform	0.264	0.244	0.257
	dichloromethane	0.265	0.245	0.258
Cl_3C-H...C_6H_8	benzene	0.263	0.244	0.258
	chloroform	0.266	0.246	0.260
	dichloromethane	0.267	0.247	0.262
Cl_3C-H...C_8H_8	benzene	0.268	0.250	0.262
	chloroform	0.270	0.252	0.265
	dichloromethane	0.271	0.253	0.265
$C_6H_5NH_2$...C_6H_6	benzene	0.394	0.390	0.386
	chloroform	0.396	0.393	0.390
	dichloromethane	0.397	0.394	0.392
$(Me_3Si)_3Si$-H…C_6F_6	benzene	-0.131 (-0.102)	-0.132 (-0.104)	-0.141 (-0.113)
	chloroform	-0.131 (-0.107)	-0.132 (-0.109)	-0.141 (-0.117)
	dichloromethane	-0.131 (-0.109)	-0.132 (-0.111)	-0.141 (-0.120)
$(Me_3Si)_3Si$-H…$C_6H_5NO_2$	benzene	-0.112	-0.115	-0.123

	chloroform	-0.115	-0.117	-0.127
	dichloromethane	-0.117	-0.119	-0.129
$(Me_3Si)_3Si$-H…C_6F_8	benzene	-0.134	-0.135	-0.141
	chloroform	-0.134	-0.134	-0.141
	dichloromethane	-0.134	-0.135	-0.141
$(Me_3Si)_3Si$-H…C_8F_8	benzene	-0.129	-0.129	-0.141
	chloroform	-0.127	-0.128	-0.139
	dichloromethane	-0.127	-0.127	-0.139
$(Me_3Si)_3Si$-H…ICF_3	benzene	-0.133	---	-0.142
	chloroform	-0.135	---	-0.144
	dichloromethane	-0.136	---	-0.145

Table S8. Principal components of the NMR shielding tensors of the hydrogen atom involved in the H-bonding.

Systems	σ_{11}^{d}	σ_{22}^{d}	σ_{33}^{d}	σ_{11}^{p}	σ_{22}^{p}	σ_{33}^{p}	σ_{11}	σ_{22}	σ_{33}	σ_{iso}
Cl_3C-H… C_5H_5N	27.88	17.84	42.53	-13.99	-0.28	-12.62	13.89	17.57	29.91	20.46
Cl_3C-H...C_6H_6	30.73	30.70	46.57	-10.00	-9.93	-5.22	20.73	20.77	41.35	27.62
Cl_3C-H...C_6H_8	31.72	28.32	43.23	-12.83	-7.83	-11.99	18.89	20.49	31.24	23.54
Cl_3C-H...C_8H_8	33.08	29.84	45.79	-15.51	-9.23	-17.49	17.57	20.61	28.30	22.16
$C_6H_5NH_2$...C_6H_6	37.05	32.03	50.83	-14.87	-1.06	-15.89	22.18	30.97	34.94	29.36
$(Me_3Si)_3Si$-H…C_6F_6	47.33	47.33	58.33	-21.02	-21.00	-16.18	26.30	26.32	42.15	31.59
$(Me_3Si)_3Si$-H…$C_6H_5NO_2$	45.25	48.46	54.13	-19.44	-22.00	-17.70	25.81	26.46	36.44	29.57
$(Me_3Si)_3Si$-H…C_6F_8	48.90	44.83	56.11	-23.94	-18.70	-20.02	24.95	26.13	36.10	29.06
$(Me_3Si)_3Si$-H…C_8F_8	51.33	47.13	57.80	-26.54	-20.22	-21.64	24.80	26.91	36.16	29.29
$(Me_3Si)_3Si$-H…ICF_3	35.33	40.73	51.16	-11.19	-13.89	-13.24	24.14	26.84	37.92	29.63
Cl_3C-H	20.02	20.02	36.52	1.91	1.91	-8.88	21.93	21.93	27.64	23.83
$(Me_3Si)_3Si$-H	33.15	33.14	48.38	-6.58	-6.55	-15.02	26.57	26.58	33.36	28.84
$C_6H_5NH_2$	20.42	27.37	43.52	0.82	-0.23	-7.81	21.24	27.14	35.72	28.04

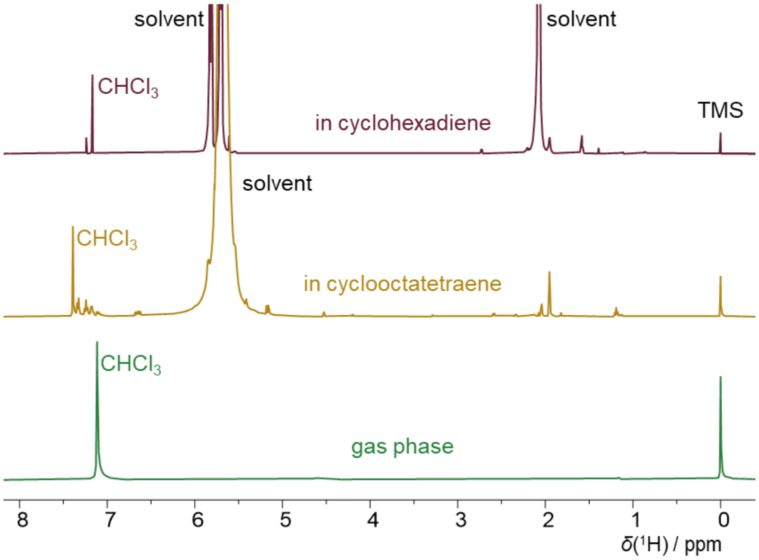

Figure S5. ^{1}H NMR spectra of chloroform in cyclohexadiene, cyclooctatetraene, and in the gas phase.

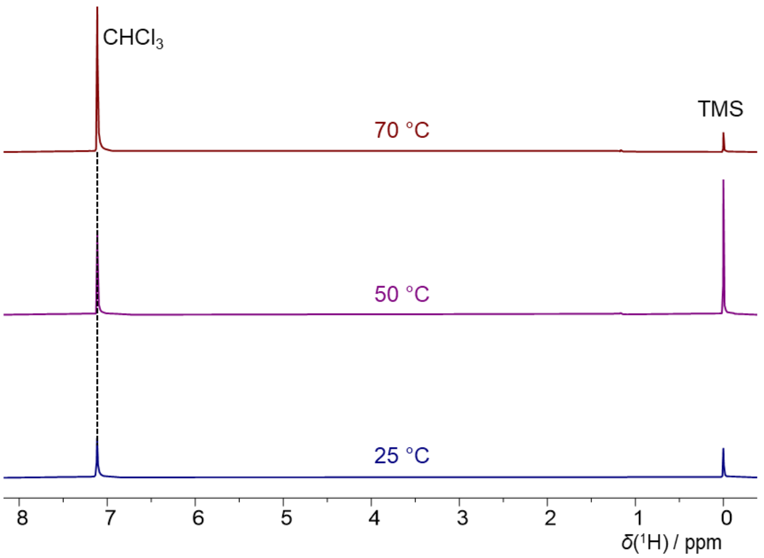

Figure S6. Variable-temperature ^{1}H NMR spectra of chloroform in $CDCl_3$.

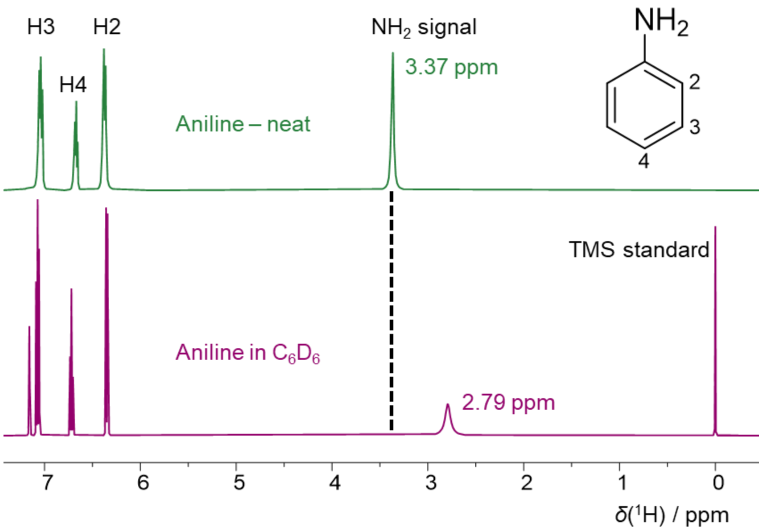

Figure S7. ^{1}H NMR spectra of aniline in the neat and in benzene.

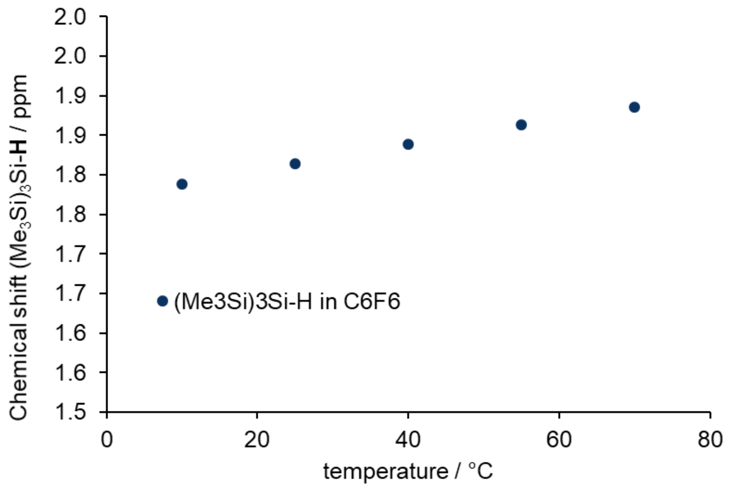

Figure S8. Temperature dependence of $(Me_3Si)_3Si–H$ proton chemical shift in hexafluorobenzene.

Methods

The NMR spectra were measured on a 600 MHz Bruker Avance III HD and 500 MHz Bruker Neo spectrometers (^{1}H at 500.3 and 600.1 MHz, respectively) in various deuterated ($CDCl_3$, C_6D_6, pyridine-d_5) and non-deuterated (cyclohexadiene, cyclooctatetraene, nitrobenzene) solvents and in the gas phase. For the solution measurements, 20 µL of the analyte was dissolved in 500 µL of the solvent. The sample temperature was calibrated using ethylene glycol. The chemical shifts were referenced to the signal of tetramethylsilane (TMS) or, to the methyl signals of $(Me_3Si)_3SiH$. The gas-phase measurements of $(Me_3Si)_3SiH$ were performed in fluorinated ethylene propylene copolymer (FEP) insert into the conventional 5-mm NMR tube.

Computational details

All molecular geometries were optimized using density functional theory (DFT) at the PBE0-D3 level[3,4] with the def2-TZVPP basis set.[5] Gibbs free energies (ΔG) were calculated within the rigid-rotor harmonic-oscillator-ideal-gas approximation at the same level of theory. The solvent effects of benzene were incorporated through the COSMO continuous solvation model during geometry optimizations.[6] NMR chemical shifts were subsequently computed for the optimized geometries using the Gauge-Including Atomic Orbital (GIAO) method.[7] Chemical shift values were computed with reference to tetramethylsilane (TMS). All calculations were performed using the Gaussian 16 software package.[8] To dissect the nuclear magnetic shielding tensors into components arising from chemical bonds and lone pairs, we performed Natural Chemical Shielding (NCS) analysis[9] using the NBO7 program[10] as integrated in the ORCA software developed by Weinhold and coworkers. The EDD were obtained using the ORCA 6.0.1 program[11] at the PBE0-D3/def2-TZVPP level of theory, employing the CPCM-C solvation model with benzene as the solvent. The results presented in Tables S2 and S3 were obtained using ORCA 6.0.1 program. The isosurface of $ICSS_{zz}$, maximum of Z-component of magnetic shielding and color-filled contour map of $ICSS_{ZZ}$ were generated using Multiwfn.[12] The

NBO orbitals and MESP values are obtained from Multiwfn program. The second-order perturbation energy and NBO charges were calculated from NBO calculations. Another levels, wB97XD/def2-TZVPP and PBE0-D3/cc-pVTZ were also used to perform the NBO calculations in different solvents (chloroform and dichloromethane).

References

1. Maier, S.; Thapa, B.; Raghavachari, K. G4 accuracy at DFT cost: unlocking accurate redox potentials for organic molecules using systematic error cancellation. *Phys.Chem.Chem.Phys*., **2020**, *22*, 4439.
2. Camaioni, D. M.; Schwerdtfeger, C. A. Comment on "Accurate Experimental Values for the Free Energies of Hydration of H^+, OH^-, and H_3O^+" *J. Phys. Chem. A.,* **2005**, *109*, 10795.
3. Adamo, C.; Barone, V. Toward reliable density functional methods without adjustable parameters: The PBE0 model. *J. Chem. Phys.* **1999,** *110*, 6158–6170.
4. Grimme, S.; Antony, J.; Ehrlich, S.; Krieg, H. A Consistentand Accurate Ab Initio Parametrization of Density Functional Dispersion Correction (DFT-D) for The 94 Elements H-Pu. *J.Chem. Phys*. **2010**, *132*, 154104.
5. Weigend, F.; Ahlrichs, R. Balanced basis sets of split valence, triple zeta valence and quadruple zeta valence quality for H to Rn: Design and assessment of accuracy, *Phys. Chem. Chem. Phys*. **2005,** *7*, 3297-3305.
6. Klamt, A.; Schüürmann, G. COSMO: a new approach to dielectric screening in solvents with explicit expressions for the screening energy and its gradient, *J. Chem. Soc. Perkin Trans.2.* **1993,** *2*, 799–805.
7. Wolinski, K.; Hinton, J.F.; Pulay, P. Efficient Implementation of the Gauge-Independent Atomic Orbital Method for NMR Chemical Shift Calculations, *J. Am. Chem. Soc.* **1990**, *112*, 8251-8260.
8. Frisch, M. J.; Trucks, G. W.; Schlegel, H. B.; Scuseria, G. E.; Robb, M. A.; Cheeseman, J. R.; Scalmani, G.; Barone, V.; Petersson, G. A.; Nakatsuji, H.; Li, X.; Caricato, M.; Marenich, A. V.; Bloino, J.; Janesko, B. G.; Gomperts, R.; Mennucci, B.; Hratchian, H. P.; Ortiz, J. V.; Izmaylov, A. F.; Sonnenberg, J. L.; Williams-Young, D.; Ding, F.; Lipparini, F.; Egidi, F.; Goings, J.; Peng, B.; Petrone, A.; Henderson, T.; Ranasinghe, D.; Zakrzewski, V. G.; Gao, J.; Rega, N.; Zheng, G.; Liang, W.; Hada, M.; Ehara, M.; Toyota, K.; Fukuda, R.; Hasegawa, J.; Ishida, M.; Nakajima, T.; Honda, Y.; Kitao, O.; Nakai, H.; Vreven, T.; Throssell, K.; Montgomery, J. A., Jr.; Peralta, J. E.; Ogliaro, F.; Bearpark, M. J.; Heyd, J. J.; Brothers, E. N.; Kudin, K. N.; Staroverov, V. N.; Keith, T. A.; Kobayashi, R.; Normand, J.; Raghavachari, K.; Rendell, A. P.; Burant, J. C.; Iyengar, S. S.; Tomasi, J.; Cossi, M.; Millam, J. M.; Klene, M.; Adamo, C.; Cammi, R.; Ochterski, J. W.; Martin, R. L.; Morokuma, K.; Farkas, O.; Foresman, J. B.; Fox, D. J. *Gaussian 16*, Revision C.01; Gaussian, Inc.: Wallingford, CT, 2016.
9. Bohmann, J. A.; Weinhold, F.; Farrar, T. C. Natural chemical shielding analysis of nuclear magnetic resonance shielding tensors from gauge-including atomic orbital calculations. *J. Chem. Phys.* **1997**, *107*, 1173-1184.
10. Glendening, E.D.; Landis, C. R.; Weinhold, F. NBO 7.0: New vistas in localized and delocalized chemical bonding theory. *J. Comput. Chem.* **2019**, *40*, 2234-2241.
11. Neese, F. Software Update: The ORCA Program System—Version 5.0. *Wiley Interdiscip Rev Comput Mol Sci* **2022**, *12* (5), e1606.
12. Lu, T. A comprehensive electron wavefunction analysis toolbox for chemists, Multiwfn. *J. Chem. Phys.* **2024**, *161*, 082503.

Optimized coordinates of the studied systems in xyz format calculated at PBE-D3/def2-TZVPP level of theory.

$CHCl_3$-Benzene

E = -1650.917986 Ha

C 1.15894100 -0.00017000 -0.00856400

H	0.07540200	-0.00389400	-0.05232300
Cl	1.74295600	-1.33449200	-1.00074900
Cl	1.63552800	-0.20816700	1.67465400
Cl	1.71892300	1.54784100	-0.63762900
C	-2.21945900	0.81474200	1.11180600
C	-2.23550400	1.37983900	-0.15717300
C	-2.24794300	0.56311900	-1.28155700
C	-2.24378300	-0.81849000	-1.13638800
C	-2.22762700	-1.38380800	0.13314000
C	-2.21563000	-0.56729000	1.25695500
H	-2.20222100	1.45130800	1.98842300
H	-2.23110300	2.45736000	-0.27062700
H	-2.25386400	1.00409200	-2.27124700
H	-2.24616500	-1.45546400	-2.01287900
H	-2.21726200	-2.46135900	0.24605200
H	-2.19466300	-1.00779700	2.24659900

Benzene

E = -232.052991 Ha

C	0.94136000	-1.02119300	0.00000100
C	1.35506600	0.30467900	-0.00000100
C	0.41371700	1.32582500	0.00000600
C	-0.94140100	1.02115500	0.00000000
C	-1.35507800	-0.30462400	-0.00000300
C	-0.41366400	-1.32584200	0.00000100
H	1.67599400	-1.81806200	-0.00000600
H	2.41250700	0.54235600	-0.00001100
H	0.73648600	2.36046900	-0.00000900
H	-1.67592700	1.81812400	-0.00000300
H	-2.41248700	-0.54244100	-0.00000200
H	-0.73657000	-2.36044200	0.00000900

$CHCl_3$

E = -1418.856927 Ha

C	0.00000000	0.00000000	0.45727800
H	0.00000000	0.00000000	1.54152400
Cl	0.00000000	1.67708100	-0.08402300
Cl	-1.45239400	-0.83854000	-0.08402300
Cl	1.45239400	-0.83854000	-0.08402300

$CHCl_3$-Pyridine

E = -1666.949764 Ha

C -1.64070300 0.02328300 0.00095700
H -0.55157400 0.12707100 0.00368600
Cl -2.07963900 -1.06088100 -1.32120200
Cl -2.34720700 1.62281000 -0.23764300
Cl -2.12876000 -0.65411800 1.55568900
C 2.26986800 1.22165500 0.00179000
C 3.65563000 1.17023300 0.00146100
C 4.27105500 -0.07192200 0.00072600
C 3.47725900 -1.20837700 0.00032500
C 2.09948600 -1.05204600 0.00069500
N 1.50057600 0.13613000 0.00144500
H 5.35158200 -0.15303500 0.00041900
H 1.75340400 2.17652000 0.00225800
H 4.23401500 2.08546600 0.00174100
H 3.91249600 -2.19966200 -0.00034200
H 1.44577100 -1.91901700 0.00004300

Pyridine

E = -248.084336 Ha

C	-1.13707100	-0.71786800	-0.00021600
C	-1.19154200	0.66845900	-0.00012600
C	0.00012300	1.37639900	0.00010800
C	1.19166200	0.66826100	0.00021300
C	1.13694300	-0.71805700	0.00011500
N	-0.00012400	-1.41024200	-0.00008900
H	0.00018600	2.46002900	0.00016400
H	-2.05313000	-1.30106400	-0.00037600
H	-2.14808900	1.17569200	-0.00024900
H	2.14831800	1.17528800	0.00034500
H	2.05289700	-1.30141500	0.00017800

$CHCl_3$-C_6H_8

E = -1652.087107 Ha

C	1.27680500	0.00875400	-0.00316000
H	0.23631400	0.04245000	0.30353500
Cl	2.25139400	0.57178200	1.35163800
Cl	1.45767700	1.06922000	-1.39921700
Cl	1.66997200	-1.65762500	-0.41988900
C	-2.21551200	-1.38510900	0.46594700
C	-2.03740300	-0.55059100	1.49556800

C	-1.90390300	0.88620700	1.27070600
C	-2.23682000	1.42245800	0.09182800
H	-2.26506200	-2.45644700	0.62742400
H	-1.95506400	-0.93095300	2.50755100
H	-1.53940900	1.51006400	2.07939600
H	-2.16093300	2.49243400	-0.06844500
C	-2.80360800	0.56379700	-0.99891500
H	-2.60354000	1.00029900	-1.97932600
H	-3.89769600	0.55318800	-0.88771600
C	-2.26825600	-0.86345500	-0.93906000
H	-1.25362900	-0.90236400	-1.36304600
H	-2.87254700	-1.51844200	-1.56991100

C_6H_8

E = -233.222394 Ha

C	-0.11077600	1.41386400	0.06385200
C	-1.25132700	0.71858300	0.11176200
C	-1.24864800	-0.72316800	-0.11195900
C	-0.10551500	-1.41418000	-0.06361500
H	-0.11376200	2.49118900	0.19068900
H	-2.19678200	1.21807100	0.29293600
H	-2.19220600	-1.22628900	-0.29295100
H	-0.10459000	-2.49164600	-0.18937400
C	1.18465200	-0.71729300	0.25162100
H	2.03368500	-1.26226200	-0.16612100
H	1.32311100	-0.72704600	1.34309500
C	1.18192600	0.72166400	-0.25170900
H	1.31949300	0.73130300	-1.34328000
H	2.02917600	1.26986400	0.16529500

Cyclooctatetraene-$CHCl_3$

E = -1728.191119 Ha

C	-1.63214800	1.54487200	-0.61136300
C	-1.69740100	1.54721600	0.72238700
C	-2.36049700	0.66510500	-1.52814000
C	-2.36051100	-0.66894000	-1.52652500
C	-2.51030300	0.66902800	1.56721400
C	-2.51027600	-0.66531500	1.56882700
C	-1.69730900	-1.54551000	0.72613600
C	-1.63211000	-1.54644800	-0.60761600
H	-1.03459700	-2.32235800	-1.08183300
H	-1.14997300	-2.32185200	1.25684700
H	-2.88529400	-1.17568200	-2.33345200
H	-3.11048600	-1.16979100	2.32291500

H	-3.11053400	1.17529200	2.32008700
H	-2.88527800	1.16990000	-2.33629300
H	-1.03467200	2.31966400	-1.08744700
H	-1.15018700	2.32493900	1.25119700
C	1.53228600	0.00004300	0.04990100
H	0.46493700	0.00016100	0.25054400
Cl	1.74072300	0.00018000	-1.69966400
Cl	2.22080100	-1.45350300	0.76947200
Cl	2.22116500	1.45328900	0.76975100

Cyclooctatetraene

E = -309.324892 Ha

C 1.68551900 -0.04758000 0.38458700
C 1.19233300 1.19233300 0.38452800
C 1.19233300 -1.19233300 -0.38452800
C -0.04758000 -1.68551900 -0.38458700
C 0.04758000 1.68551900 -0.38458700
C -1.19233300 1.19233300 -0.38452800
C -1.68551900 0.04758000 0.38458700
C -1.19233300 -1.19233300 0.38452800
H -1.73182800 -1.94920600 0.95019600
H -2.59743500 0.22704300 0.95034700
H -0.22704300 -2.59743500 -0.95034700
H -1.94920600 1.73182800 -0.95019600
H 0.22704300 2.59743500 -0.95034700
H 1.94920600 -1.73182800 -0.95019600
H 2.59743500 -0.22704300 0.95034700
H 1.73182800 1.94920600 0.95019600

$(Me_3Si)_3SiH$
E = -1517.180302 Ha

H -0.00145200 0.00156800 -2.27009200
Si -0.00013500 0.00106200 -0.76767900
Si 1.79140300 1.34112000 -0.03971700
C 3.42703400 0.43066900 -0.23498700
H 4.25726600 1.06642200 0.08618500
H 3.59859900 0.14767600 -1.27600300
H 3.44438100 -0.47985100 0.36842700
C 1.85577200 2.93291800 -1.03995000
H 2.01712100 2.72130700 -2.09935600
H 2.67419000 3.56957400 -0.69135100
H 0.92391400 3.49464800 -0.94670400
C 1.54312100 1.76371000 1.77769200
H 2.38301100 2.35738800 2.14945000
H 0.62824800 2.34266900 1.92372000

H 1.47002200 0.86014700 2.38717100
Si -2.05769400 0.87949500 -0.03948200
C -2.09267000 2.74980200 -0.24413600
H -1.31779400 3.22587200 0.36113700
H -1.92881100 3.03497700 -1.28580000
H -3.06169000 3.14873100 0.06967700
C -3.46540400 0.12950500 -1.03726800
H -3.35883200 0.36333500 -2.09892100
H -3.48704300 -0.95721000 -0.93211400
H -4.42706400 0.52430600 -0.69662200
C -2.30093300 0.46087900 1.77934600
H -1.48769300 0.85928200 2.39009500
H -2.33744600 -0.62037800 1.93161400
H -3.23998800 0.88662700 2.14423500
Si 0.26704600 -2.22031200 -0.04010400
C 0.74513500 -2.21912200 1.78069200
H 1.70086500 -1.71279700 1.93491700
H 0.84141100 -3.24426000 2.14948000
H -0.00709600 -1.70977400 2.38734300
C -1.33531500 -3.18490400 -0.24748100
H -1.19826000 -4.22387700 0.06615300
H -1.66373200 -3.18461600 -1.28927900
H -2.13454000 -2.75057000 0.35740900
C 1.62298300 -3.06558500 -1.03268800
H 2.57232900 -2.53516800 -0.93300400
H 1.36586000 -3.10009100 -2.09373200
H 1.76722800 -4.09225800 -0.68360600

$(Me_3Si)_3SiH...C_6F_6$
E = -2344.351416 Ha

H	0.34359600	0.00562100	-0.01117100
Si	-1.15313500	-0.00100900	0.00049600
Si	-1.80916300	1.66718800	-1.51744900
C	-0.79125900	1.51495400	-3.09227600
H	-0.92417200	0.53749600	-3.55952300
H	-1.08918800	2.28121200	-3.81383100
H	0.27290800	1.64482400	-2.88205200
C	-1.51616100	3.37006400	-0.77287100
H	-2.12157700	3.51812600	0.12436600
H	-1.77699900	4.15178700	-1.49205200
H	-0.46705700	3.50048900	-0.49633300
C	-3.63563400	1.47120000	-1.92535900
H	-3.83096400	0.50207500	-2.39046600
H	-4.24987700	1.54091400	-1.02447900
H	-3.96095800	2.25134400	-2.61912300
Si	-1.78245400	-2.16009000	-0.68077000

C	-0.74848800	-3.43188300	0.24235000
H	-0.88187400	-3.34406700	1.32255800
H	-1.03503800	-4.44455400	-0.05583700
H	0.31402400	-3.30374500	0.02311100
C	-1.48880300	-2.36843500	-2.52746300
H	-2.10548100	-1.67659200	-3.10532700
H	-1.73489600	-3.38685900	-2.84154300
H	-0.44276900	-2.17916200	-2.78040300
C	-3.60411900	-2.43982300	-0.30331800
H	-3.79776300	-2.36012700	0.76904400
H	-4.22977400	-1.70390500	-0.81377100
H	-3.91580300	-3.43574000	-0.63068900
Si	-1.78349100	0.47977100	2.21158400
C	-0.75951500	1.92100700	2.85415300
H	-0.89872700	2.81296800	2.23953000
H	-1.04758800	2.16546600	3.88070100
H	0.30452700	1.67415900	2.85192800
C	-1.48185200	-1.01647900	3.31179700
H	-2.09306100	-1.86590500	2.99821600
H	-1.73109600	-0.78330100	4.35099600
H	-0.43392700	-1.32389900	3.27549000
C	-3.60911500	0.93209900	2.26406000
H	-3.81083900	1.81927600	1.65881200
H	-4.22733200	0.11673600	1.88084900
H	-3.92379500	1.14315600	3.28989500
C	2.80611100	0.07047700	1.37569600
C	2.79915700	1.23694500	0.62939800
C	2.79262700	1.17314100	-0.75376600
C	2.79297800	-0.05646700	-1.39048600
C	2.79987700	-1.22272200	-0.64377200
C	2.80633600	-1.15943000	0.73929400
F	2.77661600	-2.39823500	-1.25240000
F	2.79057000	-2.27390100	1.45309300
F	2.79044300	0.13078200	2.69819400
F	2.77552300	2.41219900	1.23764600
F	2.76153200	2.28789300	-1.46728900
F	2.76287700	-0.11737500	-2.71230700

C_6F_6
E = -827.160342 Ha

C 1.38493400 0.03322600 -0.00002300
C 0.72130200 -1.18279400 0.00000400
C -0.66368900 -1.21596600 -0.00000800
C -1.38500500 -0.03325600 -0.00002500
C -0.72123900 1.18274700 0.00000700
C 0.66369900 1.21604100 -0.00001000

F -1.41038900 2.31274900 0.00006300
F 1.29779700 2.37772700 0.00002600
F 2.70814100 0.06503800 -0.00006600
F 1.41031000 -2.31277000 0.00005400
F -1.29774100 -2.37778100 0.00003500
F -2.70812100 -0.06496200 -0.00007500

$(Me_3Si)_3SiH...PhNO_2$
E = -1953.633362 Ha

H 0.14836600 0.41989500 1.03108000
Si -0.96516700 -0.00435100 0.12081200
Si -2.95102400 0.08949000 1.37685700
C -3.00149700 1.68837000 2.36502800
H -2.96966100 2.55915400 1.70669300
H -3.91864900 1.74251500 2.95882700
H -2.14984800 1.75184700 3.04610200
C -3.04585600 -1.36858300 2.56332600
H -3.05875100 -2.31649100 2.02034600
H -3.95589000 -1.31085600 3.16760000
H -2.18877100 -1.38008300 3.24060100
C -4.43193300 0.02846500 0.21673900
H -4.43445700 0.88601000 -0.46010500
H -4.42245800 -0.87951400 -0.39060700
H -5.36458900 0.04514600 0.78784900
Si -1.03571400 1.47792800 -1.70410800
C 0.69138900 1.76151900 -2.39084200
H 1.16291600 0.82132100 -2.68433900
H 0.64639100 2.41151000 -3.26967500
H 1.32586500 2.24185000 -1.64309000
C -1.75722500 3.13282100 -1.17734000
H -2.78080700 3.01914400 -0.81316700
H -1.77150000 3.82957700 -2.02049800
H -1.16146200 3.57913700 -0.37770300
C -2.11839400 0.72901600 -3.05032500
H -1.69944800 -0.21391400 -3.40991100
H -3.12808800 0.53032800 -2.68406200
H -2.19416100 1.41193300 -3.90133600
Si -0.50237900 -2.19838200 -0.58489200
C 0.09040500 -3.23095300 0.87250900
H -0.66465100 -3.27120000 1.66047000
H 0.30656500 -4.25485600 0.55361000
H 1.00209600 -2.80767500 1.29957800
C 0.83133500 -2.19740500 -1.91228400
H 0.49785900 -1.65746300 -2.80150300

H 1.07874800 -3.22103600 -2.20855400
H 1.74326300 -1.71822500 -1.54942900
C -2.06760300 -2.96967700 -1.29024700
H -2.85252300 -3.02393300 -0.53227700
H -2.45153900 -2.38586100 -2.12992200
H -1.86834700 -3.98477900 -1.64539200
C 4.37410000 -1.56091300 -0.24857500
C 3.77978100 -1.61614100 1.00588300
C 3.17847900 -0.48952800 1.54097900
C 3.18261000 0.67823700 0.79580500
C 3.76528000 0.75528000 -0.45843100
C 4.36711900 -0.37868200 -0.97781900
H 3.74309600 1.68599600 -1.00676200
H 4.82877300 -0.33879900 -1.95631100
H 4.84138700 -2.44626200 -0.66249600
H 3.78019800 -2.54106600 1.56874300
H 2.70510500 -0.50590500 2.51233000
N 2.54518000 1.87127600 1.35266000
O 2.44373000 2.84694100 0.63669400
O 2.14886100 1.82144400 2.49791800

$PhNO_2$
E = -436.443844 Ha

C 2.49979500 0.00000000 -0.00000100
C 1.80927600 1.20548200 0.00021100
C 0.42443000 1.21386300 0.00018800
C -0.24310600 0.00000000 -0.00000100
C 0.42442900 -1.21386300 -0.00019000
C 1.80927700 -1.20548200 -0.00021000
H -0.13733300 -2.13718500 -0.00032800
H 2.35019600 -2.14343100 -0.00039000
H 3.58314600 0.00000100 0.00000100
H 2.35019700 2.14343000 0.00039500
H -0.13733400 2.13718400 0.00033000
N -1.70776900 0.00000000 0.00000100
O -2.27494400 -1.07277800 0.00051800
O -2.27494300 1.07277800 -0.00051900

$C_6H_5NH_2 \ldots C_6H_6$
E = -519.440649 Ha

C 2.09956600 1.47808400 -0.05987000
C 1.64640500 0.82368600 -1.19840800
C 2.05733500 -0.47586000 -1.46079900

C 2.92068900 -1.12219200 -0.58490400
C 3.37221800 -0.46939400 0.55488900
C 2.96194400 0.83243200 0.81754900
H 1.77591800 2.49143100 0.14732200
H 0.96350500 1.32411300 -1.87428100
H 3.24010500 -2.13739700 -0.78999900
H 4.04367500 -0.97419300 1.23961700
H 3.31305300 1.34273200 1.70678100
C -1.56193500 0.87770600 0.64300300
C -2.71759200 1.39253900 0.07959600
C -3.67027500 0.55317900 -0.48469300
C -3.44380600 -0.81720400 -0.47478400
C -2.29027800 -1.34215200 0.08510700
C -1.32925700 -0.50144000 0.65516700
H -0.82488400 1.54198500 1.08053500
H -2.87420600 2.46552600 0.08166800
H -4.57267500 0.95983500 -0.92398000
H -4.17341600 -1.49007900 -0.91135600
H -2.12506700 -2.41460200 0.08668500
N -0.19932800 -1.02178700 1.26395500
H 0.06202500 -1.94192300 0.94826800
H 0.59781300 -0.40289000 1.27608700
H 1.69935900 -0.98833200 -2.34616200

Aniline
E= -287.380971 Ha

C -0.22038600 1.20124000 -0.00413700
C 1.16482600 1.19553300 0.00336300
C 1.87213600 0.00001400 0.00661300
C 1.16484500 -1.19553100 0.00332300
C -0.22035500 -1.20124900 -0.00405900
C -0.93620100 -0.00000300 -0.00877500
H -0.76106000 2.14200800 -0.00882500
H 1.69702300 2.14021300 0.00838300
H 2.95497300 0.00002400 0.01291200
H 1.69706700 -2.14019700 0.00837600
H -0.76103500 -2.14201900 -0.00859900
N -2.32011000 0.00004700 -0.07465800
H -2.76765400 -0.83531800 0.26567200
H -2.76773500 0.83494300 0.26671400

$(Me_3Si)_3SiH...C_6F_8$
E = -2543.922457 Ha

H 0.14540300 -0.08761300 0.42670900

Si 1.55705000 -0.03278900 -0.07266200
Si 2.42658000 -2.19213800 0.24425000
C 1.14067200 -3.45086800 -0.30390500
H 0.88113000 -3.31610800 -1.35609100
H 1.51788200 -4.46914100 -0.17228600
H 0.22442000 -3.34938200 0.28276900
C 2.83452100 -2.48169300 2.05792300
H 3.60920200 -1.79229000 2.40165300
H 3.19703900 -3.50257800 2.20947600
H 1.95276700 -2.33777100 2.68672200
C 3.99232500 -2.39683400 -0.77872800
H 3.78643200 -2.26172300 -1.84314700
H 4.74947900 -1.66489300 -0.48736000
H 4.41512200 -3.39576500 -0.63921300
Si 1.44077200 0.55347000 -2.34654400
C 0.17302400 1.92308200 -2.58011300
H 0.39791700 2.78783200 -1.95233900
H 0.15511700 2.25325200 -3.62281100
H -0.82772600 1.56922800 -2.32279500
C 0.93301600 -0.92697500 -3.38949700
H 1.66045600 -1.73740900 -3.30456800
H 0.86212600 -0.64328300 -4.44357900
H -0.03967100 -1.31192100 -3.07523300
C 3.13118400 1.15637600 -2.91170600
H 3.44041700 2.04028700 -2.34896500
H 3.89162900 0.38477400 -2.76985800
H 3.10928400 1.42013700 -3.97286700
Si 2.65050500 1.60040200 1.21374100
C 2.24276800 1.34215400 3.03170100
H 2.56787200 0.35691800 3.37251800
H 2.74050000 2.09753700 3.64672500
H 1.16666000 1.41955400 3.20319700
C 2.08336000 3.31870800 0.69810600
H 2.33487900 3.52003300 -0.34576100
H 2.56359600 4.08240500 1.31671500
H 1.00148700 3.42272100 0.81000400
C 4.50884700 1.45377500 0.95986400
H 4.86837000 0.47015400 1.27156700
H 4.77455200 1.59099600 -0.09085800
H 5.03878800 2.20995100 1.54586000
C -2.75809100 1.45678800 0.15331600
C -2.11066900 1.15168800 1.27162000
C -2.09244200 -0.21157900 1.80194800
C -2.57550100 -1.23602500 1.10890500
C -3.08927800 -1.04002600 -0.28456200
C -3.56993500 0.42055500 -0.56384800
F -2.53713900 -2.48224400 1.54351600

F -1.54636800 -0.36012000 2.99195000
F -1.43765200 2.04922300 1.96342200
F -2.78752400 2.67492800 -0.35390200
F -4.86490700 0.51410000 -0.17964200
F -3.53132300 0.63611300 -1.88782500
F -4.10821700 -1.87855700 -0.52806900
F -2.10871700 -1.33520700 -1.17115000

C_6F_8
E = -1026.732231 Ha

C -0.29303800 1.43112900 0.03339400
C -1.41990200 0.72852400 0.06511600
C -1.41939300 -0.72939500 -0.06519200
C -0.29207600 -1.43124100 -0.03309300
C 1.02055900 -0.74940500 0.21684300
C 1.01999800 0.75009500 -0.21685900
F -0.25512800 -2.74555100 -0.14284500
F -2.60009100 -1.29583800 -0.21655600
F -2.60103100 1.29409900 0.21642100
F -0.25689200 2.74539500 0.14357700
F 1.31338800 0.80317400 -1.53759400
F 2.00346400 1.38491200 0.43738300
F 2.00423800 -1.38355000 -0.43786700
F 1.31462000 -0.80244500 1.53734200

$(Me_3Si)_3SiH…C_8F_8$
E = -2620.020682 Ha

H 0.10693800 -0.01373300 0.29195000
Si 1.59959900 0.00473000 0.12285800
Si 2.48899400 -1.72217900 1.45083200
C 1.52950200 -3.31808100 1.19301400
H 1.53832900 -3.62033300 0.14386100
H 1.97446100 -4.12364500 1.78453600
H 0.48823700 -3.20502100 1.50049900
C 2.41237100 -1.25242000 3.27139100
H 3.00087900 -0.35425700 3.47194600
H 2.80744500 -2.06275900 3.89096200
H 1.38367100 -1.05763200 3.58349400
C 4.28535400 -2.00216400 0.96321300
H 4.36356900 -2.32285900 -0.07831100
H 4.87631200 -1.09118700 1.08016000
H 4.73146400 -2.77931400 1.59039100

Si 2.10015500 -0.33332100 -2.15186700
C 0.95108100 0.68094100 -3.24253000
H 0.97393800 1.73866900 -2.97283800
H 1.24830000 0.58825900 -4.29119800
H -0.08057200 0.33342600 -3.15424400
C 1.93226600 -2.14784700 -2.61790600
H 2.61859700 -2.76804000 -2.03688700
H 2.16227900 -2.28892900 -3.67801700
H 0.91763300 -2.50931500 -2.43855300
C 3.87693700 0.20860300 -2.45539600
H 4.00634700 1.27260700 -2.24484500
H 4.57231600 -0.34644300 -1.82158700
H 4.15461700 0.03315700 -3.49863700
Si 2.36862300 2.10414000 0.86025500
C 1.48818100 2.58139800 2.45209700
H 1.65232300 1.83589500 3.23313100
H 1.85764000 3.54425300 2.81699100
H 0.41192000 2.67027300 2.29005400
C 2.05596800 3.43262900 -0.43408200
H 2.55932400 3.19411700 -1.37369200
H 2.43281200 4.39770300 -0.08290100
H 0.98925100 3.53865600 -0.64006200
C 4.21968300 1.99881900 1.18438600
H 4.44115800 1.27127400 1.96850800
H 4.76359100 1.70088400 0.28511400
H 4.60345500 2.97117600 1.50619600
C -2.61858500 0.58109500 -1.66084800
C -2.60068000 -0.75193600 -1.60594100
C -2.16917100 1.50002100 -0.63416300
C -2.57669300 1.54353200 0.63446600
C -2.12847400 -1.57014800 -0.50700800
C -2.53352500 -1.51442900 0.76205600
C -3.55349100 -0.64098500 1.30888600
C -3.57182000 0.69086900 1.25435000
F -1.22974800 -2.48546500 -0.86158000
F -2.98035600 -1.44708300 -2.67655800
F -3.01649600 1.17542300 -2.78406300
F -1.29447300 2.40613700 -1.06441500
F -2.09056900 2.48737300 1.43866800
F -4.55546500 1.35349800 1.86014500
F -4.51946600 -1.27839800 1.96795000
F -2.01929900 -2.36853500 1.64529500

C_8F_8

E = -1102.828227 Ha

C 0.06012900 1.66938200 0.39029100
C -1.18116800 1.18116800 0.39037700
C 1.18116800 1.18116800 -0.39037700
C 1.66938200 -0.06012900 -0.39029100
C -1.66938200 0.06012900 -0.39029100
C -1.18116800 -1.18116800 -0.39037700
C -0.06012900 -1.66938200 0.39029100
C 1.18116800 -1.18116800 0.39037700
F -2.74766400 0.33445600 -1.12119900
F -2.11639000 1.78376100 1.12139200
F 0.33445600 2.74766400 1.12119900
F 1.78376100 2.11639000 -1.12139200
F 2.74766400 -0.33445600 -1.12119900
F 2.11639000 -1.78376100 1.12139200
F -0.33445600 -2.74766400 1.12119900
F -1.78376100 -2.11639000 -1.12139200

ICF_3
E = -635.179506 Ha

I	0.96957200	0.00000200	-0.00001200
C	-1.17783700	0.00001400	-0.00013000
F	-1.64158000	0.97906200	-0.76266000
F	-1.64152200	-1.15011500	-0.46643100
F	-1.64137700	0.17103300	1.22924700

$(Me_3Si)_3Si–H\cdots ICF_3$
E = -2152.367025 Ha

H	0.31625100	0.00377400	-1.11718600
Si	1.50106000	0.00417500	-0.18138600
Si	1.07704500	-1.51504600	1.56856700
C	-0.05112500	-0.76028300	2.87009100
H	-0.23511800	-1.48159000	3.67168400
H	-1.01427800	-0.47336100	2.44307800
H	0.40138300	0.12994800	3.31281800
C	0.28377500	-3.07673700	0.88626600
H	-0.67884700	-2.85380300	0.42065000
H	0.11352700	-3.79860100	1.69031500
H	0.92226300	-3.54695200	0.13528000
C	2.71975500	-1.95124600	2.37726900
H	2.55934400	-2.64161800	3.21029600
H	3.39592700	-2.43047900	1.66575600
H	3.21746000	-1.06043800	2.76748500

Si	3.34352800	-0.67859200	-1.47730800
C	3.28313800	-2.53430600	-1.77722700
H	3.32916300	-3.08689900	-0.83610900
H	2.36198000	-2.81970700	-2.29038900
H	4.12848700	-2.84756900	-2.39675600
C	3.32109200	0.21462900	-3.13135000
H	2.41385500	-0.02334100	-3.69116300
H	3.35879700	1.29742600	-2.99485100
H	4.18228300	-0.08313600	-3.73657100
C	4.92884200	-0.25038300	-0.55917600
H	4.96272000	-0.73355800	0.41979900
H	5.01315400	0.82797800	-0.40579000
H	5.80054100	-0.58045600	-1.13137000
Si	1.76732700	2.19775900	0.62693700
C	2.95659500	2.15559900	2.08452900
H	2.56214100	1.53886300	2.89535400
H	3.11678500	3.16499900	2.47395000
H	3.92663200	1.74715900	1.79226100
C	2.47443800	3.31391200	-0.71163300
H	2.57698900	4.33701400	-0.33837600
H	1.82496400	3.33649100	-1.58966700
H	3.46066200	2.96904400	-1.03066500
C	0.11269400	2.88651600	1.19327900
H	-0.32768600	2.26417200	1.97502900
H	-0.59685800	2.93440400	0.36435700
H	0.24013400	3.89715500	1.59211000
I	-2.39976600	0.00427700	-0.56383200
C	-4.52848300	-0.00753100	-0.24800400
F	-5.14403200	0.73283700	-1.16119000
F	-5.00769700	-1.24285000	-0.32344200
F	-4.82547300	0.47993100	0.95042900

Additional geometries are available at the following link:
https://github.com/rabin-uochb/JCTC-2025.git
